# Analysing the dynamic structure of warm dense matter in the imaginary-time domain: theoretical models and simulations

**DOI:** 10.1098/rsta.2022.0217

**Published:** 2023-08-21

**Authors:** Tobias Dornheim, Jan Vorberger, Zhandos A. Moldabekov, Maximilian Böhme

**Affiliations:** ^1^ Center for Advanced Systems Understanding (CASUS), D-02826 Görlitz, Germany; ^2^ Helmholtz-Zentrum Dresden-Rossendorf (HZDR), D-01328 Dresden, Germany; ^3^ Technische Universität Dresden, D-01062 Dresden, Germany

**Keywords:** warm dense matter, X-ray Thomson scattering, imaginary-time correlation functions

## Abstract

Rigorous diagnostics of experiments with warm dense matter are notoriously difficult. A key method is X-ray Thomson scattering (XRTS), but the interpretation of XRTS measurements is usually based on theoretical models that entail various approximations. Recently, Dornheim *et al.* [*Nat. Commun.* **13**, 7911 (2022)] introduced a new framework for temperature diagnostics of XRTS experiments that is based on imaginary-time correlation functions. On the one hand, switching from the frequency to the imaginary-time domain gives one direct access to a number of physical properties, which facilitates the extraction of the temperature of arbitrarily complex materials without relying on any models or approximations. On the other hand, the bulk of theoretical work in dynamic quantum many-body theory is devoted to the frequency domain, and, to the best of our knowledge, the manifestation of physics properties within the imaginary-time density–density correlation function (ITCF) remains poorly understood. In the present work, we aim to fill this gap by introducing a simple, semi-analytical model for the imaginary-time dependence of two-body correlations within the framework of imaginary-time path integrals. As a practical example, we compare our new model to extensive *ab initio* path integral Monte Carlo results for the ITCF of a uniform electron gas, and find excellent agreement over a broad range of wavenumbers, densities and temperatures.

This article is part of the theme issue ‘Dynamic and transient processes in warm dense matter’.

## 1. Introduction

The study of matter at extreme pressures ($P∼1\text{–}10^{4}\;\mathrm{MBar}$) and temperatures ($T=10^{4}\text{–}10^{8}\;K$) [1,2] constitutes a highly active frontier at the interface of a number of disciplines such as plasma physics, material science and quantum chemistry. These conditions naturally occur in a host of astrophysical objects such as giant planet interiors [3,4] and brown dwarfs [5,6]. In addition, they are important for technological applications such as the discovery of novel materials [7–9] and hot-electron chemistry [10–12]. A particularly important and topical example is given by inertial confinement fusion [13,14] as it is realized, for example, at the National Ignition Facility [15]; here, the fuel capsule is predicted to traverse the aforementioned regime on its path towards ignition [16].

From a theoretical perspective, this *warm dense matter* (WDM) can be characterized in terms of a few dimensionless parameters that are of the order of unity [17,18]: (1) the Wigner–Seitz radius, $r_{s}=d/a_{\text{B}}$, is the ratio of the average interparticle distance to the first Bohr radius, and this density parameter also serves as the quantum coupling parameter in the WDM regime [19]; (2) the degeneracy temperature, $\Theta =k_{\text{B}}T/E_{\text{F}}$, measures the thermal energy in units of the electronic Fermi energy $E_{\text{F}}$ [20], with Θ≪1 and Θ≫1 corresponding to the fully degenerate and semi-classical regimes, respectively.

Consequently, the rigorous theoretical description of WDM is notoriously difficult as there are no small parameters that can serve as the basis for a suitable expansion [21,22]. While the current great interest in the properties of WDM has sparked a surge of new developments regarding different methods [22–52], we are at present still far from having a complete understanding of the full WDM regime.

This unsatisfactory situation also represents a serious obstacle for the interpretation of experiments with WDM [53], as, because of the extreme conditions, often even basic parameters such as the temperature or the density cannot be directly measured and have to be inferred from other observations. A very important method for the diagnostics of WDM is X-ray Thomson scattering (XRTS) [54,55]; here, an X-ray beam is produced either from backlighter sources [56] or using free-electron X-ray lasers (XFELs), which have become available at large research facilities such as LCLS in the USA [57], SACLA in Japan [58] and the European XFEL in Germany [59]. An XRTS measurement gives one access to the scattering intensity signal, which is given by the convolution of the dynamic structure factor (DSF) S(q,ω) with the combined source and instrument function R(ω) [54],

1.1 I(q,ω)=S(q,ω)⊛R(ω).

Unfortunately, the numerical deconvolution of equation (1.1) is generally prevented by noise in the experimental measurement. Therefore, XRTS does not give one direct access to S(q,ω), which contains the sought-after physical information about the system of interest. The most widely used approach for the interpretation of XRTS experiments is to construct an approximate model for S(q,ω), which is then convolved with R(ω) and subsequently compared to the experimental signal I(q,ω). On the one hand, one can determine *a priori* unknown free parameters such as the temperature T by finding the best fit between theory and experiment in this way. On the other hand, the inferred parameters can depend arbitrarily strongly on the model employed for S(q,ω), which is usually based on approximations such as the widely used Chihara decomposition [55,60,61].

Recently, Dornheim *et al.* [62,63] suggested circumventing this obstacle by switching from the usual ω-representation to the imaginary-time domain. The required transformation is given by a two-sided Laplace transform

1.2 $$F(\text{q},\tau )=L[S(\text{q},\omega )]=\int_{-\infty }^{\infty }\text{d}\omega \;e^{-\omega \tau }S(\text{q},\omega ),$$

which connects the DSF to the imaginary-time density–density correlation function (ITCF) F(q,τ). The latter naturally emerges in Feynman’s imaginary-time path integral picture of statistical mechanics [64,65] and corresponds to the usual intermediate scattering function evaluated at an imaginary time t=−iℏτ, where τ∈[0,β] with the inverse temperature $\beta =1/(k_{\text{B}}T)$. Note that F(q,τ) is periodic in τ with β; see §2a.

A particular advantage of the τ-domain is given by the well-known convolution theorem,

1.3 $$L[S(\text{q},\omega )]=\frac{L[S(\text{q},\omega )⊛R(\omega )]}{L[R(\omega )]},$$

which makes the deconvolution trivial; in practice, it is easy to compute the Laplace transform of the XRTS intensity and then divide it by the Laplace transform of the instrument function R(ω). Accurate knowledge of R(ω) is usually available from source monitoring at XFEL facilities or from the characterization of backlighter emission spectra [56]. In this way, equation (1.3) gives one direct access to physical information, which enables accurate inference of the temperature of arbitrarily complex systems in thermodynamic equilibrium without the use of any model, simulation or approximation [62].

The two-sided Laplace transform defined in equation (1.2) is a unique transformation, which means that the ITCF F(q,τ) contains exactly the same information as the usual DSF S(q,ω). Indeed, it has been demonstrated in reference [64] that F(q,τ) gives one direct access to a wealth of physical information, such as the excitation energies of quasi-particles. Moreover, even complex physical processes, such as the exchange–correlation-induced alignment of pairs of electrons [66] that leads to a *roton-type* minimum in the dispersion ω(q) of the DSF, can be observed and interpreted in the τ-domain. At the same time, we note that the bulk of dynamic quantum many-body theory has been developed in the frequency domain to describe S(q,ω) and related properties.

Here, we aim to partly remedy this unsatisfactory situation by presenting a simple, semi-analytical model for the imaginary-time diffusion process. It is able to accurately capture the dependence of the ITCF on τ over a broad range of parameters. As a practical application, we consider the uniform electron gas (UEG) [17,20,67], which is the archetypical model for interacting electrons and has provided important insights in a number of different contexts. The availability of many properties of the UEG in WDM conditions [17,31,68] allows us to compare our new model—in addition to other models such as the well-known random phase approximation (RPA)—to highly accurate *ab initio* path integral Monte Carlo (PIMC) simulation results for F(q,τ).

We believe that these new insights into the imaginary-time dependence of electron–electron correlations in the WDM regime will form an important basis for future studies of real WDM applications that include both electrons and ions. This article is organized as follows. In §2, we introduce the relevant theoretical background, starting with an introduction to the PIMC method and its natural connection to the ITCF in §2a. Section 2b is devoted to a brief introduction to linear response theory, followed by a concise overview of a few important properties of the ITCF in §2c. The theoretical background is concluded by our new imaginary-time diffusion model, which is introduced in §2d. In §3, we present an extensive analysis of different properties of the ITCF, starting with a discussion of its dependence on the imaginary time τ in §3a; the subsequent §§3b and 3c focus on the wavenumber q and temperature Θ, respectively. This article concludes with a discussion and future outlook in §4.

## 2. Theory

We assume Hartree atomic units throughout. A detailed introduction to the UEG model, including the UEG Hamiltonian in different representations, can be found, for example, in references [17,20]. Note that many details about the PIMC method and the estimation of the ITCF have already been introduced elsewhere [17,64,69,70], and §2a can be skipped by readers that are familiar with these concepts.

### (a) Imaginary-time PIMC

Since its inception as a method for the description of ultracold bosonic He${\;}^{4}$ some six decades ago [71,72], the *ab initio* PIMC method [73–75] has emerged as one of the most successful tools for the description of non-ideal quantum many-body systems in thermodynamic equilibrium. Since a detailed introduction to PIMC has been presented elsewhere [76], we will here restrict ourselves to outlining the main idea and how it relates to the estimation of imaginary-time correlation functions such as F(q,τ).

As a starting point, we express the canonical partition function of $N=N^{↑}+N^{↓}$ electrons in a cubic volume $\Omega =L^{3}$ and at an inverse temperature β=1/T as

2.1 $$Z_{\beta ,N,\Omega }=\frac{1}{N^{↑}!N^{↓}!}\sum_{\sigma^{↑}\in S_{N}^{↑}}\sum_{\sigma^{↓}\in S_{N}^{↓}}\text{sgn}(\sigma^{↑},\sigma^{↓})\int_{\Omega }\;d\text{R}\;\langle \text{R}|\;e^{-\beta \hat{H}}|{\hat{\pi }}_{\sigma^{↑}}{\hat{\pi }}_{\sigma^{↓}}\text{R}\rangle ,$$

where the variable $R={\left(r_{1},\ldots ,r_{N}\right)}^{T}$ contains the coordinates of all $N^{↑}$ majority and $N^{↓}$ minority electrons. We restrict ourselves to the unpolarized (i.e. paramagnetic) case of $N^{↑}=N^{↓}=N/2$ throughout this work. The summation over all elements $\sigma^{↑,↓}$ of the respective permutation group $S_{N^{↑,↓}}$, with ${\hat{\pi }}_{\sigma^{↑,↓}}$ being the corresponding permutation operator, takes into account the antisymmetry of the partition function with respect to the exchange of particle coordinates of identical fermions. The sign function $\text{sgn}(\sigma^{↑},\sigma^{↓})$ is positive (negative) for an even (odd) number of pair exchanges in a particular combination of $\sigma^{↑}$ and $\sigma^{↓}$. Note that the corresponding bosonic partition function can be obtained by setting the sign function to unity.

Unfortunately, direct evaluation of the matrix elements of the density operator $\hat{\rho }=e^{-\beta \hat{H}}$ is precluded by the non-commutativity of the kinetic ($\hat{K}$) and potential ($\hat{V}$) contributions to the total Hamiltonian $\hat{H}=\hat{K}+\hat{V}$. A well-known way of overcoming this obstacle is based on the utilization of the exact semi-group property of the density operator, which eventually leads to

2.2 $$\begin{array}{rl} Z_{\beta ,N,\Omega } & \;=\frac{1}{N^{↑}!N^{↓}!}\sum_{\sigma^{↑}\in S_{N^{↑}}}\sum_{\sigma^{↓}\in S_{N^{↓}}}\text{sgn}(\sigma^{↑},\sigma^{↓})\\ & \;\;\times \int_{\Omega }\;d{\text{R}}_{0}\ldots \;d{\text{R}}_{P-1}\langle {\text{R}}_{0}|\;e^{-\epsilon \hat{H}}|{\text{R}}_{1}\rangle \cdots \langle {\text{R}}_{P-1}|\;e^{-\epsilon \hat{H}}|{\hat{\pi }}_{\sigma^{↑}}{\hat{\pi }}_{\sigma^{↓}}{\text{R}}_{0}\rangle . \end{array}$$

**Figure 1.**
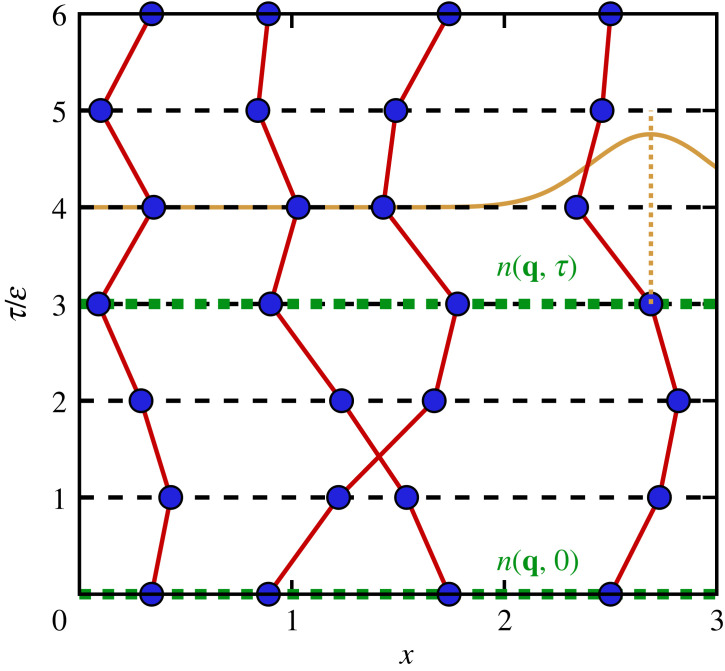
Schematic illustration of Feynman’s imaginary-time path integral formalism. Shown is a configuration X that consists of N=4 electrons on P=6 imaginary-time slices. The yellow Gaussian represents the ideal kinetic density matrix (equation (2.5)), which effectively acts as a harmonic spring between beads on adjacent slices. The dashed green lines illustrate the definition of the ITCF F(q,τ) as the correlated evaluation between densities in reciprocal space at different imaginary times, which can naturally be estimated within the PIMC formalism. Owing to the single-particle exchange of the two electrons in the centre, the configuration weight of the depicted configuration is actually negative, W(X)<0, thereby contributing to the fermion sign problem [69]. Taken from reference [64] with permission of the authors.

We have thus replaced the evaluation of a single-density matrix at the temperature T with the evaluation of P density matrices at P times the original temperature. Moreover, each corresponding exponential function can be viewed as a propagation in imaginary time by an amount of Δt=−iϵ (with ϵ=β/P); this is the origin of Feynman’s celebrated imaginary-time path integral formalism that is illustrated in figure 1. Shown is a configuration of N=4 electrons in the τ–x plane, with P=6 high-temperature factors, corresponding to P imaginary-time slices of length Δτ=ϵ. Each particle is represented by a closed path along the imaginary time. We have thus mapped the quantum system of interest onto a classical system of interacting ring polymers; this concept is often referred to as *classical isomorphism* in the literature [77]. The closed nature of the polymers is a direct consequence of the definition of the partition function as the trace of the density operator, basically leading to the same start and end points R. The situation becomes somewhat more interesting for the cases of indistinguishable quantum particles (i.e. fermions or bosons), where the application of the permutation operator ${\hat{\pi }}_{\sigma^{↑,↓}}$ in equation (2.2) leads to polymers containing more than a single particle. Such an example is depicted in figure 1, where the two particles in the centre form a single permutation cycle [78]. The presence of this single pair exchange results in a negative sign function $\text{sgn}(\sigma^{↑},\sigma^{↓})$. This is the root cause of the notorious fermion sign problem [69,79–81], the main computational bottleneck in PIMC simulations of WDM, which is discussed in more detail below.

Let us postpone the discussion of the other elements in figure 1 and return to the partition function equation (2.2). For sufficiently large P, the matrix elements of $e^{-\epsilon \hat{H}}$ can be evaluated using a suitable high-temperature approximation. For the parameters that are of interest in the present work, it is most convenient to make use of the primitive factorization $e^{-\epsilon \hat{H}}\approx e^{-\epsilon \hat{K}}\;e^{-\epsilon \hat{V}}$, which becomes exact in the limit of large P as $O(P^{-2})$ [82]. The incorporation of higher-order factorizations into PIMC simulations has been discussed extensively in the literature [83–86] and becomes advisable for lower temperatures.

In the end, one can express the partition function as

2.3 $$Z=\sum \int_{\Omega }\text{d}\text{X}\;W(\text{X}),$$

where the configuration variable $X={\left(R_{0},\ldots ,R_{P-1}\right)}^{T}$ contains the coordinates of all N particles on each of the P imaginary-time slices. Note that the symbolic notation $\sum \int_{\Omega }\text{d}X$ includes both the integration over all coordinates and the summation over all possible permutation configurations.

From a practical perspective, the partition function has been recast as a sum over all possible paths (including all permutation topologies) X, where each path contributes according to its configuration weight W(X), which is a function that we can straightforwardly evaluate. Specifically, we can factorize the weight contribution of each imaginary-time slice α into a kinetic and a potential contribution, $W_{\alpha }^{K}(X)$ and $W_{\alpha }^{V}(X)$. The latter is simply determined from the total potential energy $V(R_{\alpha })$ on slice α, and we find that

2.4 $$W_{\alpha }^{V}(\text{X})=e^{-\epsilon V({\text{R}}_{\alpha })}.$$

The kinetic part follows from the ideal single-particle density matrix for the reduced inverse temperature ϵ,

2.5 $$\rho_{0}({\text{r}}_{\alpha },{\text{r}}_{\alpha +1};\epsilon )=\lambda_{\epsilon }^{-3}\;e^{-(\pi /\lambda_{\epsilon }^{2}){\left({\text{r}}_{\alpha }-{\text{r}}_{\alpha +1}\right)}^{2}},$$

and is given by

2.6 $$W_{\alpha }^{K}(\text{X})=\prod_{l=1}^{N}\rho_{0}({\text{r}}_{l,\alpha },{\text{r}}_{l,\alpha +1};\epsilon ).$$

Returning once more to the illustration of the imaginary-time path integral picture in figure 1, we can say that the beads of the polymers interact with each other via the usual pair potential on a given time slice; the pair potential does not act between sets of coordinates for different τ. Beads of the same polymer on adjacent time slices are effectively linked via a harmonic spring potential by the ideal single-particle density matrix, equation (2.5). This is indicated by the yellow Gaussian curve on the right-hand side of figure 1. The width of the Gaussian is proportional to $\lambda_{\epsilon }=\sqrt{2\pi \epsilon }$, with ϵ=β/P. Therefore, the Gaussian becomes infinitely narrow in the classical limit of ϵ→0, where the paths are given as straight lines, corresponding to point particles in coordinate space. Conversely, the Gaussian becomes increasingly broad with decreasing temperature, which leads to more extended paths. As we shall see, this imaginary-time diffusion process decisively shapes the τ-dependence of the ITCF F(q,τ) and can thus be used to explain and interpret observations from XRTS experiments.

The basic idea of the PIMC method [76] is to randomly generate every path X with a probability that is proportional to its respective configuration weight W(X) via the Metropolis algorithm [87,88]. This is relatively straightforward for bosons and also hypothetical distinguishable particles that are sometimes referred to as Boltzmannons in the literature [89]. Indeed, modern sampling techniques such as the worm algorithm of Boninsegni *et al.* [90,91] allow efficient exploration of different permutation topologies, and exact PIMC simulations of up to $N∼10^{4}$ quantum particles are feasible. The situation dramatically changes for the case of fermions, such as the electrons in the warm dense UEG in which we are interested in the present work. In particular, the aforementioned antisymmetry of the fermionic thermal density matrix with respect to the exchange of particle coordinates implies that the configuration weight can be both positive and negative; one has W(X)<0 for the configuration depicted in figure 1. Therefore, W(X) cannot correspond to a proper probability distribution, which must be strictly non-negative. While this problem can be formally circumvented [69] by sampling the configurations proportional to the modulus weight |W(X)| and keeping track of the respective sign of W(X), it also means that the fermionic partition function Z is given as the sum over a large number of positive and negative contributions, which could cancel to a large degree. This cancellation is the origin of the fermion sign problem [69,79,80] and leads to an exponential increase in the required computation time with increasing β (i.e. decreasing the temperature T) and with increasing system size N. The sign problem is the main computational bottleneck in our simulations and limits the regime of feasible simulation parameters such as N and Θ. A detailed review of the fermion sign problem in direct PIMC simulations has been presented elsewhere [69,81] and is beyond the scope of this article.

The definition of the ITCF as the intermediate scattering function [54] evaluated at imaginary times t=−iτ is

2.7 $$F(\text{q},\tau )=\langle \hat{n}(\text{q},0)\hat{n}(-\text{q},\tau )\rangle ,$$

with τ∈[0,β]. It is easy to see that we can evaluate equation (2.7) in the path integral formalism for integer multiples of the imaginary time step ϵ by inserting the respective density operators at a distance of η∈{0,…,P−1} into equation (2.2):

2.8 $$\begin{array}{rl} F(\text{q},\tau_{j}) & \;=\frac{1}{Z_{\beta ,N,\Omega }}\frac{1}{N^{↑}!N^{↓}!}\sum_{\sigma^{↑}\in S_{N^{↑}}}\sum_{\sigma^{↓}\in S_{N^{↓}}}\text{sgn}(\sigma^{↑},\sigma^{↓})\\ & \;\;\times \int \;d{\text{R}}_{0}\ldots d{\text{R}}_{P-1}\;\langle {\text{R}}_{0}|\hat{n}(\text{q})\;e^{-\epsilon \hat{H}}|{\text{R}}_{1}\rangle \langle {\text{R}}_{1}|\;e^{-\epsilon \hat{H}}|{\text{R}}_{2}\rangle \cdots \\ & \;\;\;\cdots \langle {\text{R}}_{j}|\hat{n}(-\text{q})\;e^{-\epsilon \hat{H}}|{\text{R}}_{j+1}\rangle \cdots \langle {\text{R}}_{P-1}|\;e^{-\epsilon \hat{H}}|{\hat{\pi }}_{\sigma^{↑}}{\hat{\pi }}_{\sigma^{↓}}{\text{R}}_{0}\rangle . \end{array}$$

This is illustrated in figure 1 by the horizontal green dashed lines. In practice, F(q,τ) is obtained by computing the correlation between density operators at different imaginary times, which, in thermodynamic equilibrium, depends only on the difference of imaginary-time arguments τ. Furthermore, it directly follows from the closed nature of the paths (see figure 1) that F(q,τ) is periodic in τ with respect to β.

We conclude this subsection with a note regarding the PIMC estimation of F(q,τ) for fermions. Specifically, it is well known that the utilization of antisymmetric imaginary-time propagators alleviates the sign problem for a small number of time slices P [92–95]. Therefore, it has been shown that the combination of this idea with a higher-order factorization of the thermal density matrix allows one to obtain accurate results for parameters that are beyond the scope of direct PIMC due to the fermion sign problem [17,31]. Yet, the small number of time slices means that F(q,τ) will only be available on a sparse τ-grid. Such data can still be useful for the evaluation of τ-decay measures (equation (2.21)), which constitute the natural analogue of the dispersion relation ω(q) of the DSF in the imaginary-time domain. Yet, they will likely not suffice for the full estimation of other physical properties such as the static linear density response function χ(q) (equation (2.12)) or for the numerical inversion of equation (1.2) to reconstruct S(q,ω) via an analytic continuation [96–98]. The in-depth investigation of the application of advanced fermionic PIMC methods to the investigation of imaginary-time properties is therefore an important topic for future research.

### (b) Linear response theory

Let us next consider a modified Hamiltonian that includes an external harmonic perturbation of wavevector q and frequency ω,

2.9 $$\hat{H}={\hat{H}}_{\text{UEG}}+A\phi_{\text{ext}}(\text{q},\omega ),$$

with ${\hat{H}}_{\text{UEG}}$ being the usual UEG Hamiltonian; see references [17,99] for more details on the latter. In the limit of small perturbation amplitudes A, the response of the UEG is fully described by the linear density response function, which can be expressed as follows [20,100]:

2.10 $$\chi (\text{q},\omega )=\frac{\chi_{0}(\text{q},\omega )}{1-(4\pi /q^{2})[1-G(\text{q},\omega )]\chi_{0}(\text{q},\omega )}.$$

Here $\chi_{0}(\text{q},\omega )$ describes the density response of an ideal Fermi gas, which is sometimes known as the (temperature-dependent) Lindhard function in the literature; it can readily be computed by numerically evaluating a simple one-dimensional integral [20]. The dynamic local field correction G(q,ω) contains the full wavevector- and frequency-resolved information about electronic exchange–correlation effects; it is formally equivalent to the dynamic exchange–correlation kernel $K_{\text{xc}}(\text{q},\omega )$ that is used in the context of linear response time-dependent density functional theory simulations [101]. Setting G(q,ω)≡0 in equation (2.10) corresponds to the RPA, which describes the dynamic density response on the mean-field level. Consequently, G(q,ω) constitutes the key input for a gamut of applications such as the construction of effective potentials [102–104], quantum fluid models [48,105,106] or the construction of advanced, non-local exchange–correlation functionals for density functional theory [47,107]. The development of approximate local field corrections is an active field of research [108–120]. For the UEG, highly accurate results for G(q,ω) have been presented by Hamann *et al.* [121] based on the framework for analytic continuation of imaginary-time PIMC data developed in references [97,98,122]. First results for the static local field factor of real materials are currently being developed on the basis of either PIMC simulations [123,124] or density functional theory [125].

In the context of the present work, the main utility of χ(q,ω) is given by the fluctuation–dissipation theorem, which provides a straightforward connection between the density response and the DSF [20]:

2.11 $$S(\text{q},\omega )=-\frac{\text{Im}\chi (\text{q},\omega )}{\pi n(1-e^{-\beta \omega })}.$$

In combination with equation (1.2), any dielectric theory for the local field correction can thus be used to compute the ITCF F(q,τ).

A final interesting relation from linear response theory is the imaginary-time version of the fluctuation–dissipation theorem [64,126], which states that

2.12 $$\chi (\text{q},0)=-n\int_{0}^{\beta }\text{d}\tau \;F(\text{q},\tau ).$$

Equation (2.12) can thus be used to compute the full wavenumber dependence of the static linear density response function from a single simulation of the unperturbed system from PIMC data for F(q,τ) by solving a simple one-dimensional integral for each value of q; this approach provides the basis for a number of studies of the UEG across different regimes [19,98,127–129]. Equation (2.12) also offers a feasible way to obtain χ(q,0) from XRTS experiments. In particular, the direct evaluation of the inverse frequency sum rule

2.13 $$\left\langle \omega^{-1}\rangle =\int_{-\infty }^{\infty }\text{d}\omega \;S(\text{q},\omega )\;\omega^{-1}=-\frac{1}{2n}\chi (\text{q},0\right)$$

is usually prevented by the convolution of the DSF with the instrument function in the measured XRTS intensity. The convolution theorem for the two-sided Laplace transform (equation (1.3)), on the other hand, allows us to exactly remove the influence of R(ω) and thus opens up the way to obtain the static density response of a given system [63].

### (c) Properties of the ITCF

In thermodynamic equilibrium, the DSF obeys the detailed balance relation between positive and negative frequencies [20],

2.14 $$S(\text{q},-\omega )=S(\text{q},\omega )\;e^{-\beta \omega }.$$

Inserting equation (2.14) into the definition of the two-sided Laplace transform (equation (1.2)) gives the important symmetry relation [62,63]

2.15 $$F(\text{q},\tau )=\int_{0}^{\infty }\text{d}\omega \;S(\text{q},\omega )\{e^{-\omega \tau }+e^{-\omega (\beta -\tau )}\}=F(\text{q},\beta -\tau ).$$

In particular, Dornheim *et al.* [62] have recently shown that equation (2.15) allows for an exact, simulation-free diagnostic of the temperature of any given system from an XRTS measurement by locating the minimum of F(q,τ) at τ=β/2=1/(2T); further development of this idea is a topic of active research [63,64].

A further important set of properties related to the description of the dynamics of quantum many-body systems is given by the frequency moments of the DSF, which we define as follows:

2.16 $$M_{\alpha }=\langle \omega^{\alpha }\rangle =\int_{-\infty }^{\infty }\text{d}\omega \;S(\text{q},\omega )\;\omega^{\alpha }.$$

It is straightforward to show that all positive frequency moments can be obtained from τ-derivatives of F(q,τ) around the origin [64],

2.17 $$M_{\alpha }={\left(-1\right)}^{\alpha }{\frac{\partial^{\alpha }}{\partial \tau^{\alpha }}F(\text{q},\tau )|}_{\tau =0}.$$

We note that the odd frequency moments can alternatively be expressed in terms of nested commutator expressions [130], which are known as sum rules in the literature [131]. In the case of α=1, we have the well-known f-sum rule,

2.18 $$M_{1}=\langle \omega^{1}\rangle =\frac{{\text{q}}^{2}}{2}.$$

An important insight into the physical origin of the observed τ-dependence of F(q,τ) can be obtained from the spectral representation of the DSF, which is given by [20]

2.19 $$S(\text{q},\omega )=\sum_{m,l}P_{m}\|n_{ml}(\text{q}){\|}^{2}\delta (\omega -\omega_{lm}).$$

Equation (2.19) states that the DSF is the sum over all possible transitions between the eigenstates l and m (with $\omega_{lm}$ being the energy difference) of the full N-body Hamiltonian, where $P_{m}$ is the occupation probability of the initial state and $\|n_{ml}(\text{q}){\|}^{2}$ denotes the corresponding transition matrix element. Inserting equation (2.19) into equation (1.2) then gives [64]

2.20 $$F(\text{q},\tau )=\sum_{m,l}P_{m}\|n_{ml}(\text{q}){\|}^{2}\;e^{-\tau \omega_{lm}}.$$

In other words, the τ-decay is shaped by the energy difference between the eigenstates for transitions that are important at a particular wavevector q. More specifically, large energy differences as they occur in the non-collective single-particle regime where the dispersion relation of the DSF is $\omega (q)∼q^{2}$ lead to a pronounced τ-decay, with F(q,τ) almost vanishing around the minimum at τ=β/2. Conversely, energetically low-lying transitions such as the roton feature of the UEG [66,132] manifest as a reduced τ-decay; see references [63,64] for an extensive discussion of this effect. In other words, the existence of a low-energy excitation is equivalent to the stability of density correlations throughout the imaginary time. Dornheim *et al.* [64] recently suggested quantifying this mechanism via a relative decay measure of the form

2.21 $$\Delta F_{\tau }(\text{q})=\frac{F(\text{q},0)-F(\text{q},\tau )}{F(\text{q},0)},$$

which we will also employ in the present work.

### (d) Single-particle delocalization model

To gain further insight into the physical meaning of F(q,τ), we will present a simple model for its τ-dependence. It has recently been reported [133] based on extensive, spin-resolved PIMC results for the ITCF that the τ-dependence is almost exclusively due to single-particle imaginary-time diffusion effects; density–density correlations between electrons of different spin-orientation are nearly unaffected by the thermal delocalization of the paths. On the basis of this empirical observation, we decompose the total ITCF into

2.22 F(q,τ)=S(q)+ΔF(q,τ),

where the static structure factor (SSF) corresponds to the τ→0 limit of the ITCF, S(q)=F(q,0). The full τ-dependence is thus, by definition, contained in the function ΔF(q,τ)=F(q,τ)−F(q,0).

As the next step, we consider the imaginary-time diffusion of a single particle at the inverse temperature β in a volume $\Omega =L^{3}$, with $L\gg \lambda_{\beta }$. In this case, the appropriate imaginary-time propagator is simply given by the diagonal elements of the ideal thermal density matrix $\rho_{0}(r,r;\beta )$; see equation (2.5). To estimate the corresponding single-particle ITCF $F_{\text{SP}}(\text{q},\tau^{\prime })$, we insert a single intermediate time slice at $\tau^{\prime }$. This can be expressed as

2.23 $$\rho_{0}(\text{r},\text{r};\beta )=\int_{\Omega }\text{d}{\text{r}}^{\prime }\;\rho_{0}(\text{r},{\text{r}}^{\prime };\tau^{\prime })\rho_{0}({\text{r}}^{\prime },\text{r};\beta -\tau^{\prime }).$$

Inserting the corresponding density operators $\hat{n}(\text{q})$ and $\hat{n}(-q)$ at the appropriate imaginary times and performing a few straightforward transformations then gives a simple expression for the single-particle ITCF:

2.24 $$F_{\text{SP}}(\text{q},\tau^{\prime })=\int_{\Omega }\text{d}\Delta \text{r}\;P(\Delta \text{r},\tau^{\prime })\;\text{cos}(\text{q}\cdot \Delta \text{r}),$$

with Δr being the displacement between the position of the particle at τ=0 (which is the same as at τ=β owing to the diagonal nature of the trace, leading to closed trajectories in the path integral picture) and $\tau =\tau^{\prime }$. The probability of a particular displacement is given by the three-dimensional Gaussian distribution

2.25 $$P(\Delta \text{r},\tau )={\left(\frac{\lambda_{\beta }}{\lambda_{\tau }\lambda_{\beta -\tau }}\right)}^{3}\text{exp}\left(-\pi \frac{\lambda_{\beta }^{2}}{\lambda_{\tau }^{2}\lambda_{\beta -\tau }^{2}}\Delta {\text{r}}^{2}\right),$$

which takes into account the distance in imaginary-time to the time slice of interest starting both from τ=0 and from τ=β.

**Figure 2.**
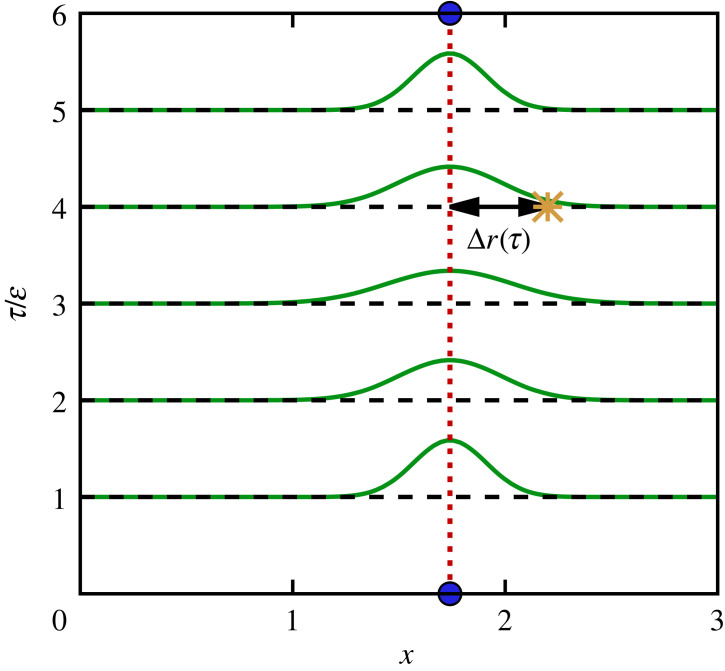
Schematic illustration of the single-particle diffusion through the imaginary time and its estimation via equation (2.24). The green curves show the Gaussian probability distribution equation (2.25) of a diffused particle coordinate at a difference τ from its origin (blue beads), and the yellow star represents a particular realization.

The basic idea behind equation (2.24) is schematically illustrated in figure 2*a*. The blue beads correspond to the start and end points at τ=0 and τ=β, which are always equal. The green curves depict the Gaussian probability distributions from equation (2.25) for different values of τ; they are symmetric with respect to τ=β/2. In other words, the symmetry relation (2.15) of the ITCF not only can be seen as a consequence of the detailed balance of the DSF but also naturally emerges as a consequence of the β-periodicity within the imaginary-time path integral formalism. The yellow star at τ=4ϵ indicates a particular realization of the displacement Δr(τ), which is weighted with the probability P(Δr,τ). The final expectation value for $F_{\text{SP}}(\text{q},\tau )$ then follows from an integral over all possible values of Δr, which we evaluate numerically.

**Figure 3.**
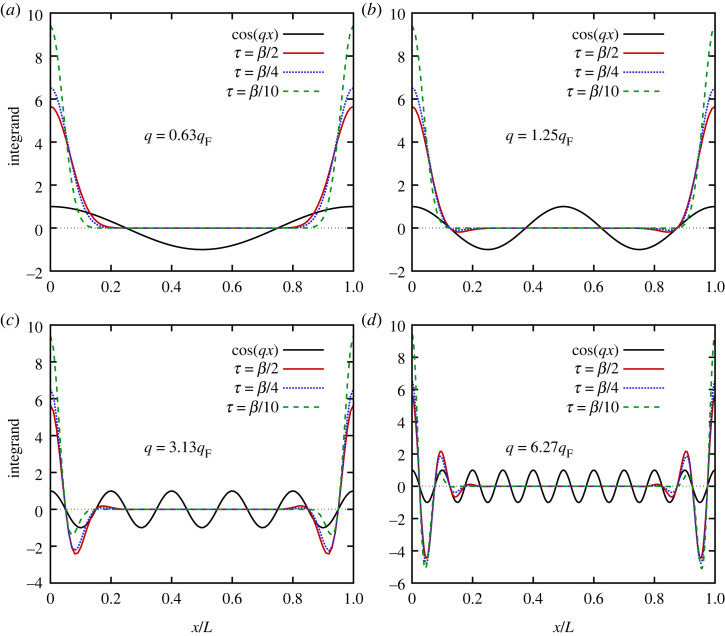
Integrand of the estimator for the single-particle ITCF $F_{\text{SP}}(\text{q},\tau )$, equation (2.24), for N=34 and Θ=1 for (*a*) $q=0.63q_{\text{F}}$, (*b*) $q=1.25q_{\text{F}}$, (*c*) $q=3.13q_{\text{F}}$ and (*d*) $q=6.27q_{\text{F}}$. The solid black sinusoidal lines show the respective cosine functions, and the solid red, dotted blue and dashed green curves represent the full integrands for τ=β/2, τ=β/4 and τ=β/10, respectively.

In figure 3, we analyse the integrand of equation (2.24), which is depicted along the x-component of Δr at y=z=0 for N=34 and Θ=1. We note that such single-particle expressions do not, by definition, depend on the density parameter $r_{s}$ for a given value of Θ. The different panels of figure 3 correspond to different wavevectors $q={\left(q,0,0\right)}^{T}$, and the sinusoidal black lines show the cosine from equation (2.24). Figure 3a has been obtained for the minimum possible wavenumber q=2π/L, figure 3b for $q=2q_{\text{min}}$, figure 3c for $q=5q_{\text{min}}$ and figure 3d for $q=10q_{\text{min}}$. In addition, the solid red, dotted blue and dashed green lines show the products of the cosine and the thermal probability function P(Δr,τ) for different values of τ. Note that we employ standard periodic boundary conditions such that x→x+L for x<0. Evidently, the Gaussian becomes increasingly narrow with decreasing τ, since the corresponding path has only a shorter imaginary-time interval to diffuse away from its position at τ=0 in that case. In the limit of τ→0, P(Δr,τ) becomes a delta distribution. Consequently, the single-particle ITCF attains unity for τ=0 and τ=β for all q.

**Figure 4.**
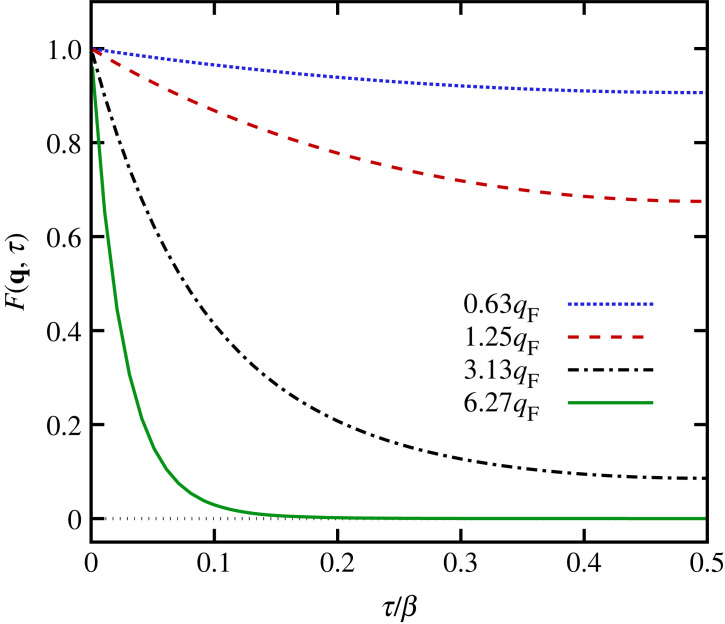
Single-particle ITCF (equation (2.24)) for different wavevectors q for N=34 and Θ=1.

For $q=q_{\text{min}}$, the cosine is positive over the entire x-range where the thermal distribution has significant values. Consequently, the oscillating nature of the cosine function has a small impact, as it is almost constant in the relevant x-interval. Still, the small decay of the cosine function for x≤L/2 becomes more important with increasing τ≤β/2, since the thermal distribution is spread out to larger x in this case. This is the origin of the comparably small τ-decay of the corresponding single-particle ITCF, which is depicted as the dotted blue line in figure 4.

In figure 3*b*, we show the integrand of equation (2.24) for a moderate wavevector, $q=1.25q_{\text{F}}$. In this case, the cosine function oscillates twice as fast as for $q=0.63q_{\text{F}}$, which has a noticeable impact on the integrand. Indeed, the latter even becomes slightly negative for x≳L/8; the resulting cancellation becomes more pronounced with increasing τ, which explains the larger τ-decay of the corresponding single-particle ITCF (dashed red curve) in figure 4. Further increasing the wavenumbers to $q=3.13q_{\text{F}}$ and $q=6.27q_{\text{F}}$ leads to the integrands shown in figure 3*c*,*d*, which exhibit an even more substantial cancellation of positive and negative contributions. Consequently, the τ-decay of $F_{\text{SP}}(\text{q},\tau )$, shown in figure 4 as the dash–dotted black and solid green curves, is even sharper. The sharp decay in the single-particle limit of $q\gg q_{\text{F}}$ is thus the result of competition between the delta distribution limit of the thermal distribution equation (2.25) for τ→0 and the cancellation of the integrand as a consequence of the rapidly oscillating cosine function for large q at any finite value of τ.

## 3. Results

All PIMC results were obtained using the extended ensemble approach introduced in reference [70], which is a canonical adaptation of the worm algorithm of Boninsegni *et al.* [90,91]. We typically employ P=200 imaginary-time slices, which is sufficient with respect to the convergence of the factorization error [98] and sufficient to resolve F(q,τ) on a useful τ-grid.

### (a) Dependence on the imaginary time

**Figure 5.**
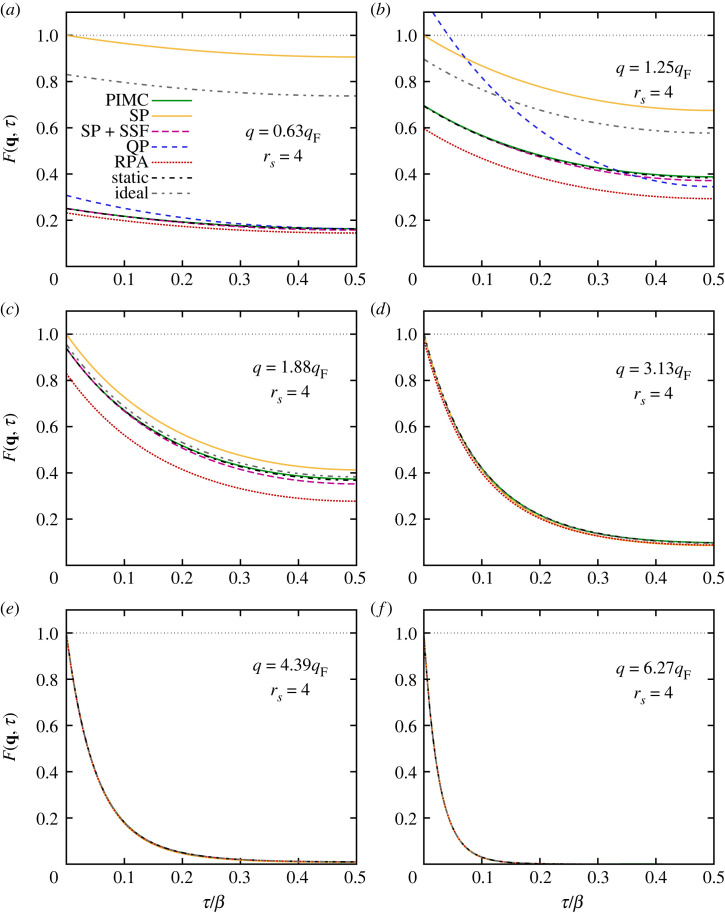
Imaginary-time dependence of the ITCF F(q,τ) for different wavenumbers q for the unpolarized UEG with N=34, $r_{s}=4$ and Θ=1. Solid green: exact PIMC results; solid yellow: single-particle ITCF $F_{\text{SP}}(\text{q},\tau )$, equation (2.24); long-dashed purple: combination of SSF with single-particle ITCF, equation (2.22); dashed blue: quasi-particle (QP) expansion, equation (3.2); dotted red: RPA; dash–dotted black: static approximation, i.e. $G_{\text{static}}(\text{q},\omega )\equiv G(\text{q},0)$; dash–double-dotted grey: ideal Fermi gas.

We begin our in-depth analysis of the τ-dependence of the ITCF of the warm dense UEG at the electronic Fermi temperature (i.e. Θ=1) for a metallic density of $r_{s}=4$, which is shown in figure 5. It is sufficient to show F(q,τ) in the half-range of 0≤τβ/2, since all curves are symmetric around τ=β/2; see equation (2.15) above. The solid green line shows our exact PIMC results; we note that the statistical uncertainty of these data is smaller than the width of the curve and can be neglected here. The dash–dotted black curve depicts the *static approximation* [97] and was obtained by setting $G_{\text{static}}(\text{q},\omega )\equiv G(\text{q},0)$ in equation (2.10). For completeness, we note that we have used the neural-net parametrization of $G(\text{q},\omega =0;r_{s},\theta )$ from reference [127], but the analytic parametrization of the local field factor [134] within the recently introduced effective static approximation [45] would have worked equally well for this purpose. The static approximation provides a very accurate description of F(q,τ) for all depicted wavevectors and over the entire τ-range. This is consistent with previous investigations of the DSF S(q,ω), which have reported the same trend [97]. The only deviations that are visible on the depicted scale appear for the intermediate wavenumbers of $q=1.25q_{\text{F}}$ and $q=1.88q_{\text{F}}$ that are shown in figure 5b,c, but the systematic errors are very small.

**Figure 6.**
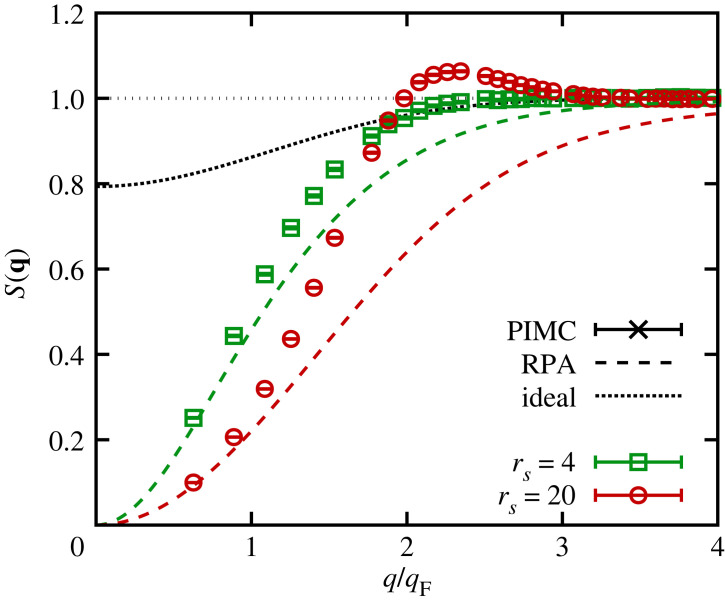
PIMC results for the SSF of the UEG with N=34 at Θ=1 for $r_{s}=4$ (green squares) and $r_{s}=20$ (red circles). The dashed curves show corresponding results within the RPA, and the dotted line depicts the SSF of the ideal Fermi gas, which is independent of $r_{s}$.

Let us next consider the dotted red curve, which corresponds to the RPA. It is well known that the RPA becomes exact in the limit as q→0, where the UEG is dominated by the collective plasmon feature; see equation (3.1). For $q=0.63q_{\text{F}}$, which is the smallest wavenumber that is accessible for simulations with N=34 particles, one is already within the pair continuum [66], and the DSF substantially differs from the plasmon shape with respect to both its peak position and the peak shape. In this regime, the RPA becomes increasingly inaccurate, and the dotted red curve noticeably deviates from the solid green PIMC results. With increasing q, the systematic error of the RPA becomes more pronounced and attains a maximum around intermediate wavenumbers, $q∼1.5q_{\text{F}}$. In the single-particle limit of $q\gg q_{\text{F}}$, the RPA becomes exact again, and no systematic deviations are visible for $q=4.39q_{\text{F}}$ and $q=6.27q_{\text{F}}$ (figure 5*e*,*f*). Interestingly, the main source of error in the RPA at intermediate wavenumbers seems to be a constant shift in F(q,τ) that does not depend on the imaginary time. In fact, it is easy to see that the first derivative of F(q,τ) around the origin is exactly reproduced by the RPA, since it fulfils the f-sum rule; cf. equations (2.17) and (2.18). From equation (2.22), it is then clear that the bulk of the RPA error should already be present in the SSF S(q), which is shown in figure 6. The green squares show our exact PIMC results for $r_{s}=4$, and the green dashed line represents the corresponding RPA. Evidently, the RPA underestimates the true magnitude of S(q) for intermediate q, which explains the observed trends in figure 5.

The dashed blue curves in figure 5 were computed from a simple quasi-particle ansatz. In this model, it is assumed that the DSF consists of a single delta-like peak around the quasi-particle excitation energy $\omega_{\text{QP}}$,

3.1 $$S_{\text{SP}}(\text{q},\omega )=A\{\delta (\omega -\omega_{\text{QP}})+e^{-\beta \omega_{\text{QP}}}\delta (\omega +\omega_{\text{QP}})\},$$

and the second term on the right-hand side corresponds to the respective contribution at negative frequencies and obeys the detailed balance relation equation (2.14). Inserting equation (3.1) into the two-sided Laplace transform equation (1.2) then gives the corresponding ITCF,

3.2 $$F_{\text{QP}}(\text{q},\tau )=A\{e^{-\tau \omega_{\text{QP}}}+e^{-(\beta -\tau )\omega_{\text{QP}}}\}.$$

For the case of a plasmon excitation in the UEG, we have $\omega_{\text{QP}}=\omega_{\text{p}}$ (with $\omega_{\text{p}}=\sqrt{3/r_{s}^{3}}$ being the usual plasma frequency [20]) for q→0, and the normalization A of equations (3.1) and (3.2) is determined by the SSF S(q)=F(q,0),

3.3 $$A=\frac{S(\text{q})}{1+e^{-\beta \omega_{\text{QP}}}}.$$

In the limit of small wavevectors, the latter can be computed analytically from the parabolic expansion [135]

3.4 $$S(\text{q})=\frac{q^{2}}{2\omega_{\text{p}}}\text{coth}\left(\frac{\beta \omega_{\text{p}}}{2}\right).$$

As a final ingredient in computing the quasi-particle ITCF $F_{\text{QP}}(\text{q},\tau )$, we require some information about the wavenumber dependence of the excitation energy, which can be expanded as [136]

3.5 $$\frac{\omega^{2}(q)}{\omega_{\text{p}}^{2}}=1+B_{2}(r_{s},\Theta ){\left(\frac{q}{q_{\text{F}}}\right)}^{2}$$

within the RPA in the limit of small q. We note that the coefficient $B_{2}(r_{s},\Theta )$ has been parametrized by Hamann *et al.* [136]. For $q=0.63q_{\text{F}}$, equation (3.2) gives the correct qualitative description of F(q,τ), but it substantially overestimates the static limit of τ→0; this is a direct consequence of the overestimation of the true SSF by the q→0 expansion given in equation (3.4). For $q=1.25q_{\text{F}}$, the validity of this simple plasmon approximation breaks down, and neither the τ-decay nor the static limit is described accurately. Therefore, we omit the corresponding results from the other panels for even larger values of q.

Let us next consider the results for the semi-analytical single-particle model $F_{\text{SP}}(\text{q},\tau )$ that we introduced in §2d, which is depicted by the solid yellow curves in figure 5. We find that $F_{\text{SP}}(\text{q},\tau )$ exhibits the correct qualitative τ-decay for all q. Yet it always exhibits a static limit of $F_{\text{SQ}}(\text{q},0)\equiv 1$, which leads to large systematic errors for small q. From a physical perspective, this deficiency can be attributed to the lack of screening effects in the underlying single-particle imaginary-time diffusion process. Combining these results for the thermal delocalization with the exact description of static correlation effects (i.e. by using our PIMC results for S(q)=F(q,τ)) via equation (2.22) leads to the long-dashed purple curves. Clearly, equation (2.22) leads to a spectacular improvement over the bare $F_{\text{SP}}(\text{q},\tau )$ for $q≲2q_{\text{F}}$, i.e. in the regime where the SSF S(q) has not yet reached the single-particle limit of S(q→∞)=1. This finding constitutes one of the central observations of the present work, as it implies that the complicated dynamic behaviour of the quantum UEG can be accurately approximated by a phenomenologically simple combination of static correlations, which can easily be estimated from equilibrium simulations, with the simple Gaussian imaginary-time diffusion of a single particle.

**Figure 7.**
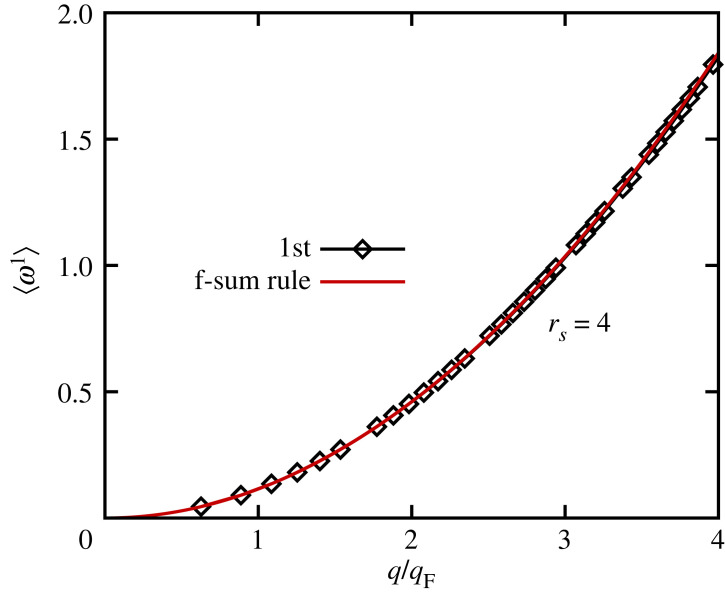
First frequency moment of the UEG at $r_{s}=4$ and Θ=1. Solid red: exact f-sum rule, equation (2.18); black diamonds: numerical derivative of the single-particle ITCF $F_{\text{SP}}(\text{q},\tau )$, as given by equation (2.17).

In fact, we can show that the τ-dependence of the single-particle ITCF, $\Delta F_{\text{SP}}(\text{q},\tau )$, does become exact in the limit as τ→0 by numerically evaluating the derivative of $F_{\text{SP}}(\text{q},\tau )$ with respect to τ around the origin. The results are shown as the black squares in figure 7 and compared to the exact f-sum rule (equation (2.18)), which is depicted by the solid red line via equation (2.17). We find perfect agreement between the numerical results obtained from the single-particle ITCF and the exact sum rule for all q. Returning once more to figure 5, we find that $F_{\text{SP}}(\text{q},\tau )$ even becomes exact for large wavenumbers where S(q)→1, and we find perfect agreement with the PIMC reference data for $q=6.27q_{\text{F}}$.

Finally, the grey dash–double-dotted curves in figure 5 show results for the ITCF of the ideal Fermi gas [137], $F_{0}(\text{q},\tau ),$ and we observe similar, though not the same, trends as in $F_{\text{SP}}(\text{q},\tau )$. In particular, fermionic exchange effects lead to density correlations between electrons of the same spin-orientation, and the static limit $F_{0}(\text{q},0)$ that is depicted as the dotted black curve in figure 6 deviates from unity for $q≲2q_{\text{F}}$. Therefore, $F_{0}(\text{q},\tau )$ is somewhat closer to the PIMC results for $q≲2q_{\text{F}}$ than is $F_{\text{SP}}(\text{q},\tau )$, even though they exhibit nearly identical dependence on the imaginary time.

**Figure 8.**
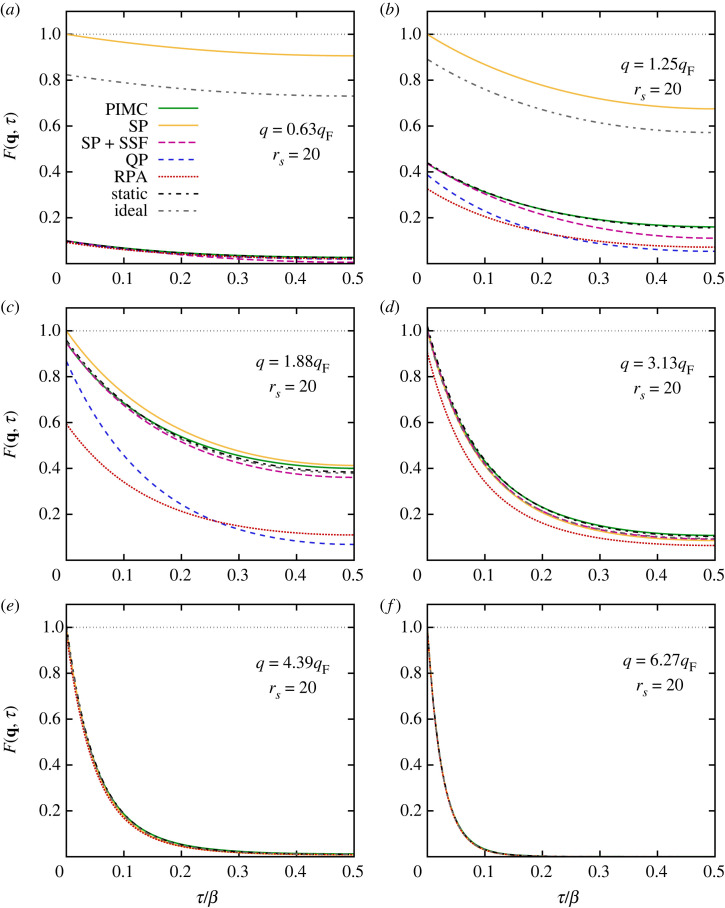
Imaginary-time dependence of the ITCF F(q,τ) for different wavenumbers q for the unpolarized UEG with N=34, $r_{s}=20$ and Θ=1. Solid green: exact PIMC results; solid yellow: single-particle ITCF $F_{\text{SP}}(\text{q},\tau )$, equation (2.24); long-dashed purple: combination of SSF with single-particle ITCF, equation (2.22); dashed blue: quasi-particle (QP) expansion, equation (3.2); dotted red: RPA; dash-dotted black: static approximation, i.e. $G_{\text{static}}(\text{q},\omega )\equiv G(\text{q},0)$; dash–double-dotted grey: ideal Fermi gas.

We next repeat this analysis at a lower density, $r_{s}=20$, the results of which are shown in figure 8. These conditions are characterized by the onset of strong electronic correlations and constitute the boundary to the strongly coupled electron liquid regime [128]. As a consequence, the dispersion relation of the DSF exhibits a pronounced roton feature [97,138], which has recently been explained by Dornheim *et al.* [66] in terms of a new pair alignment model. Since we find overall similar trends of F(q,τ) to those for $r_{s}=4$, we here restrict ourselves to a brief discussion of the main differences between the two regimes. The most pronounced difference comes from the static limit F(q,0)=S(q), which is shown in figure 6 as the red circles and red dashed line for PIMC and RPA. We find that S(q) attains smaller values than in the $r_{s}=4$ case for $q≲2q_{\text{F}}$, which is directly reflected by our results for F(q,τ) in figure 8. In addition, the RPA is less accurate as electronic exchange–correlation effects are substantially more important in the electron liquid regime. Surprisingly, we find that the RPA is even less accurate than the bare single-particle ITCF $F_{\text{SP}}(\text{q},\tau )$ or the corresponding function of the ideal Fermi gas $F_{0}(\text{q},\tau )$ for intermediate wavenumbers; this is a direct consequence of the overestimation of the Coulomb repulsion in the RPA, which is discussed in more detail in reference [66], for example.

In addition, the combination of our new single-particle imaginary-time diffusion model for $F_{\text{SP}}(\text{q},\tau )$ with the SSF via equation (2.22) overall exhibits a comparable accuracy to that for $r_{s}=4$. An exception occurs only for the smallest depicted wavenumber, $q=0.63q_{\text{F}}$, where the corresponding long-dashed purple curve exhibits a too-pronounced decay towards τ=β/2. Finally, we find exactly the same functional form of all models for F(q,τ) as for $r_{s}=4$ at $q=6.27q_{\text{F}}$, as electron–electron correlations do not influence density correlations in the single-particle regime.

### (b) Dependence on the wavenumber

**Figure 9.**
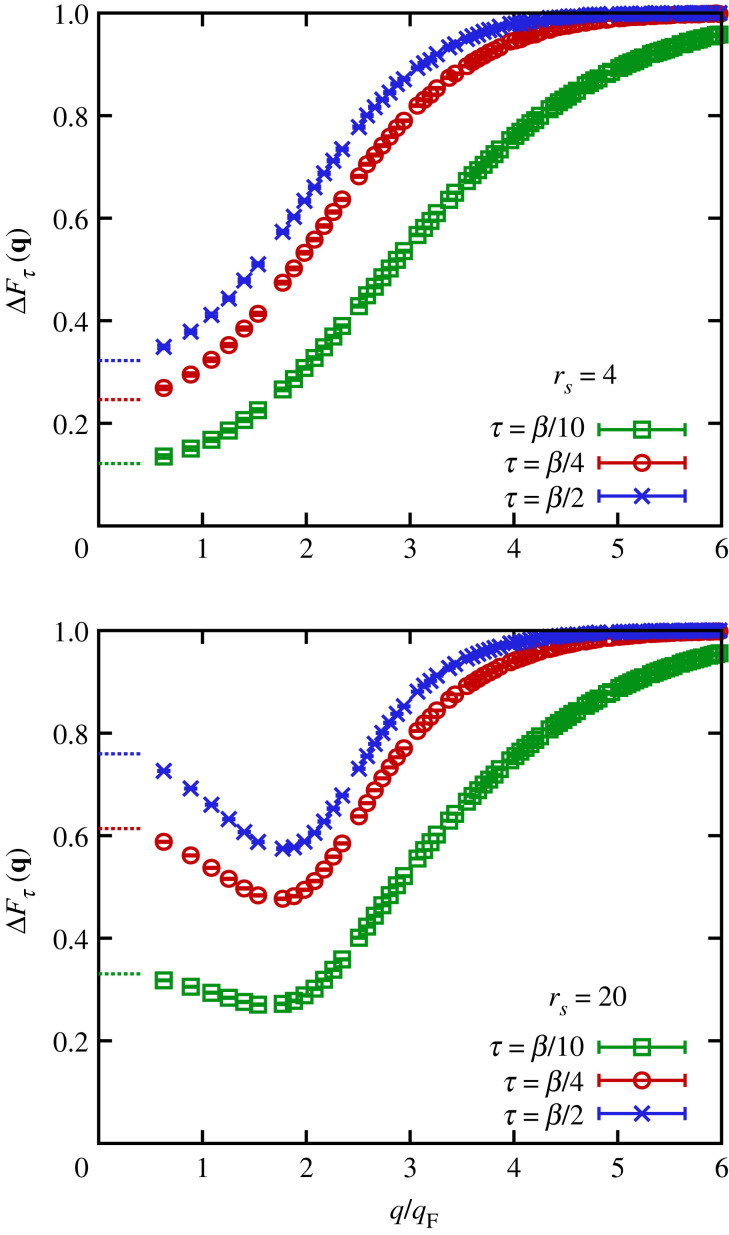
*Ab initio* PIMC results for the relative decay measure $\Delta F_{\tau }(\text{q})$ for τ=β/2 (blue crosses), τ=β/4 (red circles) and τ=β/10 (green squares) of the UEG at Θ=1 for $r_{s}=4$ (top) and $r_{s}=20$ (bottom).

To gain better qualitative insight into the dependence of imaginary-time density–density correlations on the wavenumber q, Dornheim *et al.* [64] recently suggested analysing the relative decay measure $\Delta F_{\tau }(\text{q})$ defined in equation (2.21). The results are shown in figure 9 for $r_{s}=4$ (top) and $r_{s}=20$ (bottom) based on exact PIMC simulation data for F(q,τ). More specifically, the green squares, red circles and blue crosses show $\Delta F_{\tau }(\text{q})$ for τ=β/10, τ=β/4 and τ=β/2, respectively. For $r_{s}=4$, the three depicted datasets exhibit qualitatively similar behaviour. In the limit of small q, they approach the exact plasmon quasi-particle limit that directly follows from $F_{\text{QP}}(\text{q}\to 0,\tau )$; see equation (3.2). Upon increasing q, all three curves monotonically increase and eventually attain the single-particle limit of $\Delta F_{\tau }(\text{q}\to \infty )=1$ for large q; this is a direct consequence of the steeper decay of F(q,τ) with τ for 0≤τ≤β/2 that is depicted in figures 5*f* and 8*f*. Furthermore, it is easy to see that the onset of the single-particle limit happens for increasingly large q for decreasing imaginary-time distances τ in $\Delta F_{\tau }(\text{q})$.

**Figure 10.**
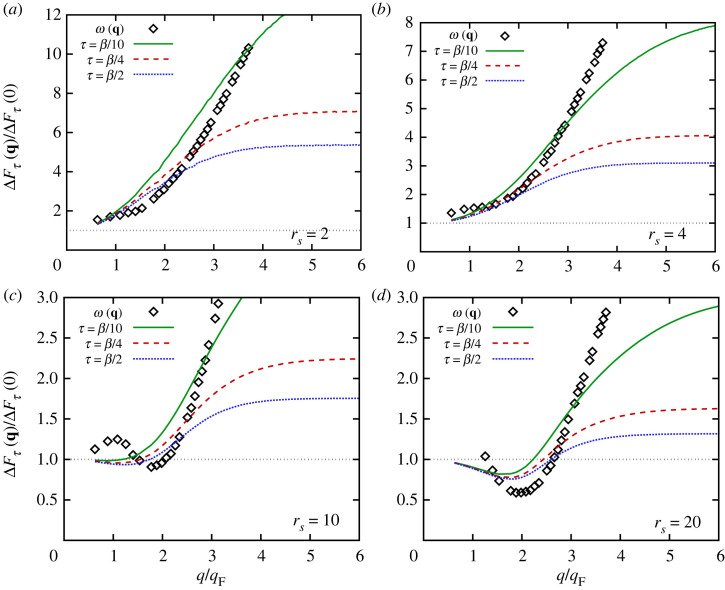
PIMC results for the dispersion relation ω(q) of the DSF (black diamonds, taken from reference [97]) and relative decay measure of the ITCF $\Delta F_{\tau }(\text{q})$ for τ=β/2 (dotted blue), τ=β/4 (dashed red) and τ=β/10 (solid green) for the unpolarized UEG with N=34 at Θ=1 for different values of the density parameter $r_{s}$. All curves have been renormalized to their respective q→0 limits, given by the plasma frequency $\omega_{\text{p}}$ in the case of ω(q).

For $r_{s}=20$, the relative deviation measure exhibits an even more interesting dependence on q. While again all three datasets attain their respective plasmon limits for q→0, $\Delta F_{\tau }(\text{q})$ has a minimum for intermediate wavenumbers of $q∼2q_{\text{F}}$ for all depicted values of τ; for larger q, we then recover the same single-particle limit as for $r_{s}=4$. From a physical perspective, the minimum in the relative decay measure signals a reduced decay of correlations along the imaginary time; in other words, electronic correlations remain more stable throughout the imaginary-time diffusion process described in §2d in the strongly coupled case of $r_{s}=20$. Indeed, Dornheim *et al.* [64] have suggested that such a reduced τ-decay of F(q,τ) can directly be translated to a shift of spectral weight in the DSF towards lower frequencies; this effect manifests as a *roton-type* minimum in the dispersion relation ω(q) of S(q,ω); cf. figure 10. This effect appears when the wavelength $\lambda_{q}=2\pi /q$ is comparable to the average interparticle distance d. It is explained in reference [66] in terms of the spontaneous alignment of pairs of electrons, which was further substantiated in the subsequent work [132].

The connection between the relative measure of τ-decay $\Delta F_{\tau }(\text{q})$ and the dispersion ω(q) of S(q,ω) is investigated in more detail in figure 10, where we overlay both quantities at Θ=1 for different values of the density parameter $r_{s}$; note that each curve has been rescaled by its respective q→0 limit, which is given by the plasma frequency $\omega_{\text{p}}$ in the case of the dispersion relation. For the metallic density of $r_{s}=2$, the PIMC-based results for ω(q) taken from reference [97] exhibit a smooth, monotonic crossover between the collective regime, where the DSF corresponds to a sharp plasmon feature (equation (3.1)), and the single-particle regime with $\lambda_{q}\ll d$, where $\omega (\text{q})∼q^{2}$. The corresponding results for the decay measure $\Delta F_{\tau }(\text{q})$ exhibit very similar behaviour; the main difference from ω(q) arises from the fact that the single-particle limit is given by a constant instead of a parabolic divergence.

For $r_{s}=4$, we find a very similar picture to that of $r_{s}=2$ for both ω(q) and the three datasets for $\Delta F_{\tau }(\text{q})$. Upon further decreasing the density to $r_{s}=10$, the dispersion starts to exhibit the incipient *roton minimum* around intermediate wavenumbers. This effect is qualitatively reflected in the relative τ-decay measure $\Delta F_{\tau }(\text{q})$ as a minimum around $q∼2q_{\text{F}}$, although its overall behaviour is less complicated than that of ω(q), as it does not exhibit the local maximum of the latter around $q=q_{\text{F}}$.

Finally, we show results for the strongly coupled case of $r_{s}=20$ in figure 10*d*. In this case, both the roton minimum in ω(q) and the reduced decay in $\Delta F_{\tau }(\text{q})$ are more pronounced, as expected. Interestingly, the physical mechanism of the roton—the spontaneous alignment of pairs of electrons [66]—manifests in $\Delta F_{\tau }(\text{q})$ with a similar magnitude for all three considered values of τ. This finding is of practical importance for the consideration of different approximate theories for F(q,τ), which are discussed next.

**Figure 11.**
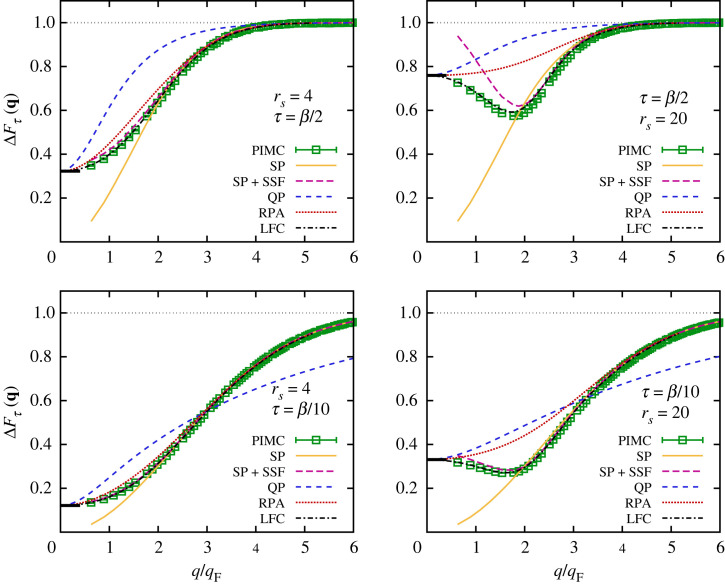
Relative imaginary-time decay measure $\Delta F_{\tau }(\text{q})$ (equation (2.21)) of the unpolarized UEG at $r_{s}=4$ (left) and $r_{s}=20$ (right) for Θ=1. The top and bottom rows correspond to τ=β/2 and τ=β/10. Green squares: *ab initio* PIMC results; solid yellow: single-particle ITCF model $F_{\text{SP}}(\text{q},\tau )$ (equation (2.24)); long-dashed purple: single-particle τ-decay combined with PIMC results for the SSF S(q) via equation (2.22); dashed blue: quasi-particle ITCF $F_{\text{QP}}(\text{q},\tau )$ (equation (3.2)); dotted red: RPA; dash–dotted black: static approximation using the local field correction (LFC) from reference [127] as input.

In figure 11, we show the wavenumber dependence of $\Delta F_{\tau }(\text{q})$ for both $r_{s}=4$ (left column) and $r_{s}=20$ (right column) for τ=β/2 (top row) and τ=β/10 (bottom row). The green squares are results obtained from our *ab initio* PIMC simulations and provide an unassailable benchmark for the other depicted theoretical approaches.

The dashed blue curves were computed from the quasi-particle approximation for the ITCF, $F_{\text{QP}}(\text{q},\tau )$, defined in equations (3.2) and (3.5). For $r_{s}=4$, the corresponding decay measure qualitatively follows the PIMC data; the limits of both large and small wavenumbers are correctly reproduced, although this happens only in the limit for very large q in the case of τ=β/10. In addition, $\Delta F_{\tau }(\text{q})$ exhibits a smooth and monotonic transition between these collective and single-particle limits, which is phenomenologically correct but quantitatively strongly deviates from the green squares. In particular, we find that while $F_{\text{QP}}(\text{q},\tau )$ becomes, by definition, exact in the plasmon limit as q→0, the change in the position of the delta peak described by equation (3.5) does not constitute a good first-order correction to this limit. In other words, the finite width of the true DSF for q>0 appears to be more important than the plasmon shift away from the plasma frequency $\omega_{\text{p}}$. This trend can be seen particularly well for the more strongly coupled case of $r_{s}=20$, where the true $\Delta F_{\tau }(\text{q})$ has a negative slope for small q; in contrast, the quasi-particle approximation $F_{\text{QP}}(\text{q},\tau )$ exhibits the opposite behaviour for both depicted values of τ.

The full RPA is represented by the dotted red curves in figure 11. It is exact in the limits of both q→0 and $q\gg q_{\text{F}}$, and represents a substantial improvement over the simple quasi-particle ansatz in between. For $r_{s}=4$, the RPA performs comparably well over the entire q-range, with a maximum relative error around intermediate wavenumbers $q∼2q_{\text{F}}$. In addition, we note that the τ-decay measure within the RPA is more accurate for τ=β/10 than for τ=β/2. This can be explained by the fact that while the SSF S(q)=F(q,0) in the RPA exhibits systematic errors for intermediate **q** (see figure 6), it does attain the correct first derivative given by the well-known f-sum rule (equation (2.18)). For $r_{s}=20$, the qualitative accuracy of the RPA noticeably deteriorates as electron–electron exchange–correlation effects become more important. In particular, the RPA, too, does not reproduce the negative slope of the exact PIMC data for $\Delta F_{\tau }(\text{q})$ for small q. This is consistent with the inability of the RPA to capture the roton minimum in the dispersion relation of the DSF, which has been investigated in detail in references [66,97].

This systematic shortcoming of the mean-field-based RPA is remedied by including exact PIMC results for the static local field correction via equation (2.10), and the results for $\Delta F_{\tau }(\text{q})$ are shown as the dash–dotted black curve in figure 11. For a metallic density of $r_{s}=4$, no systematic deviations between the static approximation and the PIMC reference results can be resolved on the depicted scale. For the electron liquid at $r_{s}=20$, too, we find that including the static local field correction leads to a spectacular improvement, and the dash–dotted black curve nicely captures the roton minimum of $\Delta F_{\tau }(\text{q})$ around $q=2q_{\text{F}}$. At the same time, small deviations from the green squares can be resolved in particular for τ=β/2 in this regime; yet the effect is significantly smaller than the corresponding underestimation of the true depth of the roton minimum of S(q,ω) due to the static approximation, which is discussed in more detail in reference [66].

Let us conclude this analysis by considering our new single-particle imaginary-time diffusion model $F_{\text{SP}}(\text{q},\tau )$ (see equation (2.24) in §2d), which is included in figure 11 as the solid yellow curve. Evidently, this simple, semi-analytical model becomes exact in the single-particle regime of $q\geq 3q_{\text{F}}$ but does not reproduce either the collective plasmon limit or the roton minimum for intermediate q at $r_{s}=20$. The combination of $F_{\text{SP}}(\text{q},\tau )$ with the correct static structure F(q,0)=S(q) via equation (2.22) (see the long-dashed purple curves in figure 11) substantially mitigates this deficiency. More specifically, our model is very accurate at $r_{s}=4$ over the entire q-range. Indeed, no systematic errors can be resolved on the depicted scale for the smaller value of the imaginary time τ=β/10, since equation (2.22) becomes exact for τ=0 both with respect to F(q,0) and with respect to the first slope, which is given by the f-sum rule that has been investigated in detail in figure 7. Even in the more complicated case of $r_{s}=20$, the combination of the correct static structure with the simple single-particle model for the imaginary-time diffusion discussed in §2d is capable of reproducing the roton minimum of $\Delta F_{\tau }(\text{q})$ with remarkable precision; it only breaks down for $q≲q_{\text{F}}$, where it fails to attain the true plasmon limit in the collective regime. For τ=β/10, even this flaw is basically removed, and the long-dashed purple curve gives a very good description of the entire wavenumber range.

### (c) Dependence on the temperature

**Figure 12.**
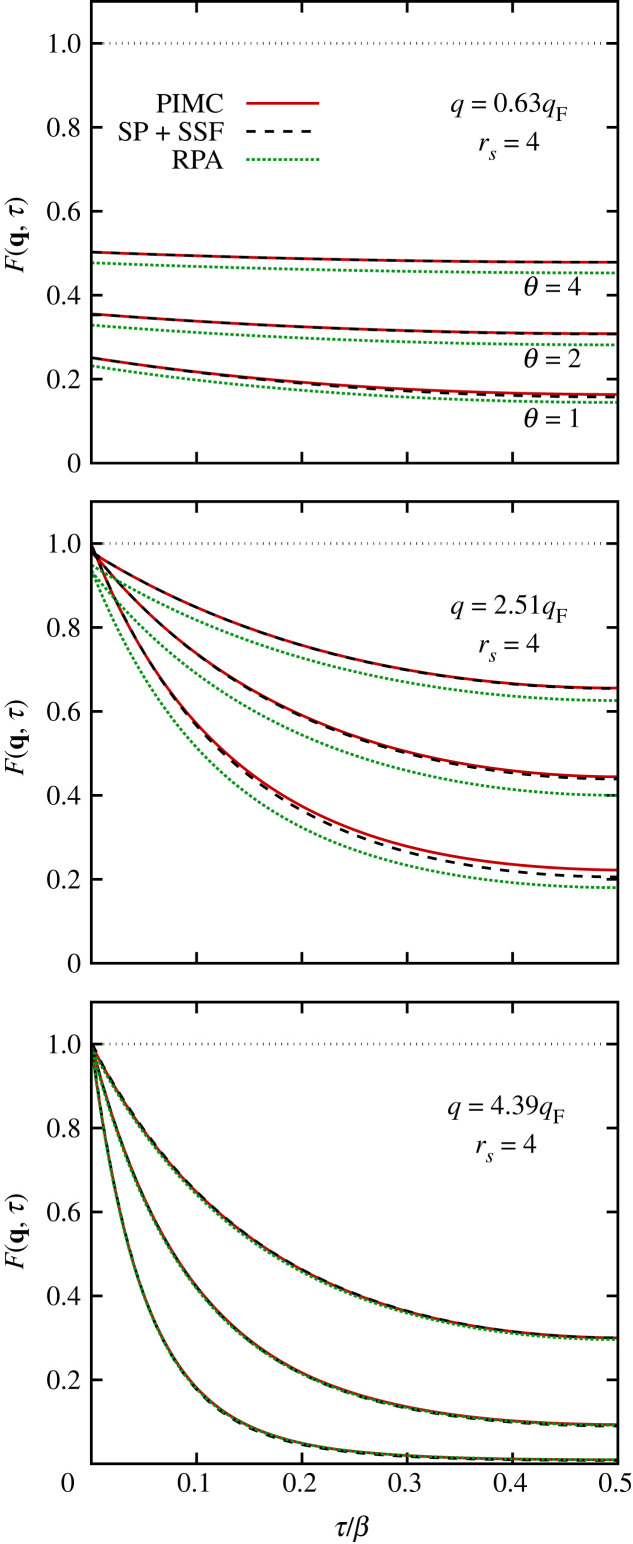
ITCF for different Θ. Solid red: PIMC; dotted green: RPA; dashed black: combination of $F_{\text{SP}}(\text{q},\tau )$ with S(q) via equation (2.22).

We conclude this investigation of the ITCF of the warm dense UEG by briefly touching upon its dependence on the temperature. In figure 12, we show F(q,τ) at $r_{s}=4$ for $q=0.63q_{\text{F}}$ (top), $q=2.51q_{\text{F}}$ (centre) and $q=4.39q_{\text{F}}$ (bottom). The solid red lines correspond to exact PIMC simulation data for Θ=4, Θ=2 and Θ=1, and the curves are ordered with respect to temperature as indicated in figure 12a. Overall, we find a similar trend for all depicted wavenumbers: the RPA (dotted green) underestimates the static limit of F(q,0)=S(q) but exhibits the correct qualitative decay with respect to τ. The combination of our new single-particle model for the imaginary-time diffusion and the exact static limit via equation (2.22) overall fits even better to the PIMC reference data and captures the correct trends for all depicted temperatures and wavenumbers. In fact, it becomes more accurate with increasing temperature, which can be understood intuitively in the following way. As we have previously shown in this work, equation (2.22) becomes exact in the limit of τ→0 with respect to both F(q,0) and the slope at the origin. For higher temperatures, the imaginary-time propagation described in §2d is carried out over a shorter distance in the τ-domain. Therefore, systematic errors have a shorter distance to accumulate, which explains the increased relative accuracy for larger values of Θ.

## 4. Discussion

In this work, we have introduced a simple, semi-analytic model that gives new insights into the dependence of the ITCF F(q,τ) on the imaginary time τ. In practice, we find that the combination of the static structure S(q)=F(q,0) with the simple single-particle imaginary-time diffusion function $F_{\text{SP}}(\text{q},\tau )$ (equation (2.22)) gives a highly accurate description of the ITCF over a broad range of densities, temperatures and wavenumbers. Remarkably, our model is even capable of capturing the roton feature of the strongly coupled electron liquid [64,66,97], which emerges from the exchange–correlation-induced alignment of pairs of electrons. Even in this regime, the effect of electron–electron correlations on the imaginary-time diffusion is relatively small.

In addition, our model also very accurately captures the behaviour of the ITCF with respect to the temperature parameter Θ and generally agrees well with the PIMC results for the decay measure $\Delta F_{\tau }(\text{q})$.

We believe that our new results represent an important first step towards a more thorough theoretical understanding of dynamic quantum many-body effects formulated in imaginary time. Such an improved understanding will be of immediate use for the interpretation of *ab initio* PIMC simulations of WDM [17,123,124,127], which give direct access to the ITCF. While previous direct PIMC simulations have mostly been restricted to the UEG, future efforts will produce results for the ITCF of real materials such as hydrogen [123,124]. In addition, we note that previous models for S(q,ω) such as the Chihara decomposition [60,61], but also time-dependent density functional theory calculations [36,44], can easily be translated to the imaginary-time domain and benchmarked against upcoming PIMC results. In addition, having an improved understanding of the physical properties of F(q,τ) will be helpful for the interpretation of XRTS experiments with WDM. As mentioned earlier, the latter give straightforward access to the ITCF [62] and contain the same physical information as the DSF. Performing the corresponding analysis of the observed XRTS signal in the τ-domain, therefore, has the potential to yield physical insights beyond temperature diagnostics without any models or approximations [64].

## Data Availability

This article has no additional data.

## References

[1] Fortov VE. 2009 Extreme states of matter on Earth and in space. Phys.-Usp **52**, 615-647. (10.3367/UFNe.0179.200906h.0653)

[2] Drake R 2018 High-energy-density physics: foundation of inertial fusion and experimental astrophysics. Graduate Texts in Physics. Cham, Switzerland: Springer International Publishing.

[3] Benuzzi-Mounaix A *et al.* 2014 Progress in warm dense matter study with applications to planetology. Phys. Scripta **T161**, 014060. (10.1088/0031-8949/2014/t161/014060)

[4] Vorberger J, Tamblyn I, Militzer B, Bonev SA. 2007 Hydrogen-helium mixtures in the interiors of giant planets. Phys. Rev. B **75**, 024206. (10.1103/PhysRevB.75.024206)

[5] Saumon D, Hubbard WB, Chabrier G, van Horn HM. 1992 The role of the molecular-metallic transition of hydrogen in the evolution of Jupiter, Saturn, and brown dwarfs. Astrophys. J **391**, 827-831. (10.1086/171391)

[6] Becker A, Lorenzen W, Fortney JJ, Nettelmann N, Schöttler M, Redmer R. 2014 Ab initio equations of state for hydrogen (H-REOS.3) and helium (He-REOS.3) and their implications for the interior of brown dwarfs. Astrophys. J. Suppl. Ser **215**, 21. (10.1088/0067-0049/215/2/21)

[7] Kraus D *et al.* 2016 Nanosecond formation of diamond and lonsdaleite by shock compression of graphite. Nat. Commun. **7**, 10970. (10.1038/ncomms10970) 26972122 PMC4793081

[8] Kraus D *et al.* 2017 Formation of diamonds in laser-compressed hydrocarbons at planetary interior conditions. Nat. Astron. **1**, 606-611. (10.1038/s41550-017-0219-9)

[9] Lazicki A *et al.* 2021 Metastability of diamond ramp-compressed to 2 terapascals. Nature **589**, 532-535. (10.1038/s41586-020-03140-4) 33505034

[10] Brongersma ML, Halas NJ, Nordlander P. 2015 Plasmon-induced hot carrier science and technology. Nat. Nanotechnol. **10**, 25-34. (10.1038/nnano.2014.311) 25559968

[11] Waldecker L, Bertoni R, Ernstorfer R, Vorberger J. 2016 Electron-phonon coupling and energy flow in a simple metal beyond the two-temperature approximation. Phys. Rev. X **6**, 021003. (10.1103/PhysRevX.6.021003)

[12] Ernstorfer R, Harb M, Hebeisen CT, Sciaini G, Dartigalongue T, Miller RJD. 2009 The formation of warm dense matter: experimental evidence for electronic bond hardening in gold. Science **323**, 1033-1037. (10.1126/science.1162697) 19164708

[13] Betti R, Hurricane OA. 2016 Inertial-confinement fusion with lasers. Nat. Phys. **12**, 435-448. (10.1038/nphys3736)

[14] Zylstra AB *et al.* 2022 Burning plasma achieved in inertial fusion. Nature **601**, 542-548. (10.1038/s41586-021-04281-w) 35082418 PMC8791836

[15] Moses EI, Boyd RN, Remington BA, Keane CJ, Al-Ayat R. 2009 The national ignition facility: ushering in a new age for high energy density science. Phys. Plasmas **16**, 041006. (10.1063/1.3116505)

[16] Hu SX, Militzer B, Goncharov VN, Skupsky S. 2011 First-principles equation-of-state table of deuterium for inertial confinement fusion applications. Phys. Rev. B **84**, 224109. (10.1103/PhysRevB.84.224109)

[17] Dornheim T, Groth S, Bonitz M. 2018 The uniform electron gas at warm dense matter conditions. Phys. Rep. **744**, 1-86. (10.1016/j.physrep.2018.04.001) 29341671

[18] Ott T, Thomsen H, Abraham JW, Dornheim T, Bonitz M. 2018 Recent progress in the theory and simulation of strongly correlated plasmas: phase transitions, transport, quantum, and magnetic field effects. Eur. Phys. J. D **72**, 84. (10.1140/epjd/e2018-80385-7)

[19] Dornheim T, Vorberger J, Moldabekov Z, Röpke G, Kraeft WD. 2022 The uniform electron gas at high temperatures: ab initio path integral Monte Carlo simulations and analytical theory. High Energy Density Phys. **45**, 101015. (10.1016/j.hedp.2022.101015)

[20] Giuliani G, Vignale G. 2008 Quantum theory of the electron liquid. Cambridge, UK: Cambridge University Press.

[21] Graziani F, Desjarlais MP, Redmer R, Trickey SB (eds). 2014 Frontiers and challenges in warm dense matter. Cham, Switzerland: Springer International Publishing.

[22] Bonitz M, Dornheim T, Moldabekov ZA, Zhang S, Hamann P, Kählert H, Filinov A, Ramakrishna K, Vorberger J. 2020 Ab initio simulation of warm dense matter. Phys. Plasmas **27**, 042710. (10.1063/1.5143225)

[23] Benedict LX *et al.* 2012 Molecular dynamics simulations and generalized Lenard-Balescu calculations of electron-ion temperature equilibration in plasmas. Phys. Rev. E **86**, 046406. (10.1103/PhysRevE.86.046406) 23214699

[24] Driver KP, Militzer B. 2012 All-electron path integral Monte Carlo simulations of warm dense matter: application to water and carbon plasmas. Phys. Rev. Lett. **108**, 115502. (10.1103/PhysRevLett.108.115502) 22540485

[25] Brown EW, Clark BK, DuBois JL, Ceperley DM. 2013 Path-integral Monte Carlo simulation of the warm dense homogeneous electron gas. Phys. Rev. Lett. **110**, 146405. (10.1103/PhysRevLett.110.146405) 25167016

[26] Sjostrom T, Daligault J. 2014 Gradient corrections to the exchange-correlation free energy. Phys. Rev. B **90**, 155109. (10.1103/PhysRevB.90.155109)

[27] Whitley HD, Scullard CR, Benedict LX, Castor JI, Randles A, Glosli JN, Richards DF, Desjarlais MP, Graziani FR. 2015 Lenard-Balescu calculations and classical molecular dynamics simulations of electrical and thermal conductivities of hydrogen plasmas. Contrib. Plasma Phys. **55**, 192-202. (10.1002/ctpp.201400066)

[28] Ping Y *et al.* 2015 Differential heating: a versatile method for thermal conductivity measurements in high-energy-density matter. Phys. Plasmas **22**, 092701. (10.1063/1.4929797)

[29] Karasiev VV, Calderin L, Trickey SB. 2016 Importance of finite-temperature exchange correlation for warm dense matter calculations. Phys. Rev. E **93**, 063207. (10.1103/PhysRevE.93.063207) 27415377

[30] Zhang S, Wang H, Kang W, Zhang P, He XT. 2016 Extended application of Kohn-Sham first-principles molecular dynamics method with plane wave approximation at high energy—from cold materials to hot dense plasmas. Phys. Plasmas **23**, 042707. (10.1063/1.4947212)

[31] Dornheim T, Groth S, Malone FD, Schoof T, Sjostrom T, Foulkes WMC, Bonitz M. 2017 Ab initio quantum Monte Carlo simulation of the warm dense electron gas. Phys. Plasmas **24**, 056303. (10.1063/1.4977920) 27768371

[32] Schöttler M, Redmer R. 2018 Ab initio calculation of the miscibility diagram for hydrogen-helium mixtures. Phys. Rev. Lett **120**, 115703. (10.1103/PhysRevLett.120.115703) 29601747

[33] Karasiev VV, Sjostrom T, Dufty J, Trickey SB. 2014 Accurate homogeneous electron gas exchange-correlation free energy for local spin-density calculations. Phys. Rev. Lett. **112**, 076403. (10.1103/PhysRevLett.112.076403) 24579621

[34] Desjarlais MP, Scullard CR, Benedict LX, Whitley HD, Redmer R. 2017 Density-functional calculations of transport properties in the nondegenerate limit and the role of electron-electron scattering. Phys. Rev. E **95**, 033203. (10.1103/PhysRevE.95.033203) 28415190

[35] Grabowski P *et al.* 2020 Review of the first charged-particle transport coefficient comparison workshop. High Energy Density Phys. **37**, 100905. (10.1016/j.hedp.2020.100905)

[36] Baczewski AD, Shulenburger L, Desjarlais MP, Hansen SB, Magyar RJ. 2016 X-ray Thomson scattering in warm dense matter without the Chihara decomposition. Phys. Rev. Lett. **116**, 115004. (10.1103/PhysRevLett.116.115004) 27035307

[37] Zhang S *et al.* 2018 Theoretical and experimental investigation of the equation of state of boron plasmas. Phys. Rev. E **98**, 023205. (10.1103/PhysRevE.98.023205) 30253522

[38] Zhang S *et al.* 2019 Equation of state of boron nitride combining computation, modeling, and experiment. Phys. Rev. B **99**, 165103. (10.1103/PhysRevB.99.165103)

[39] Ping Y *et al.* 2019 Heat-release equation of state and thermal conductivity of warm dense carbon by proton differential heating. Phys. Rev. E **100**, 043204. (10.1103/PhysRevE.100.043204) 31771018

[40] Zhang S *et al.* 2020 Benchmarking boron carbide equation of state using computation and experiment. Phys. Rev. E **102**, 053203. (10.1103/PhysRevE.102.053203) 33327061

[41] Karasiev VV, Dufty JW, Trickey SB. 2018 Nonempirical semilocal free-energy density functional for matter under extreme conditions. Phys. Rev. Lett. **120**, 076401. (10.1103/PhysRevLett.120.076401) 29542959

[42] Ramakrishna K, Dornheim T, Vorberger J. 2020 Influence of finite temperature exchange-correlation effects in hydrogen. Phys. Rev. B **101**, 195129. (10.1103/PhysRevB.101.195129)

[43] Militzer B, González-Cataldo F, Zhang S, Driver KP, Soubiran F. 2021 First-principles equation of state database for warm dense matter computation. Phys. Rev. E **103**, 013203. (10.1103/PhysRevE.103.013203) 33601631

[44] Ramakrishna K, Cangi A, Dornheim T, Baczewski A, Vorberger J. 2021 First-principles modeling of plasmons in aluminum under ambient and extreme conditions. Phys. Rev. B **103**, 125118. (10.1103/PhysRevB.103.125118)

[45] Dornheim T, Cangi A, Ramakrishna K, Böhme M, Tanaka S, Vorberger J. 2020 Effective static approximation: a fast and reliable tool for warm-dense matter theory. Phys. Rev. Lett. **125**, 235001. (10.1103/PhysRevLett.125.235001) 33337174

[46] Ding YH, White AJ, Hu SX, Certik O, Collins LA. 2018 Ab initio studies on the stopping power of warm dense matter with time-dependent orbital-free density functional theory. Phys. Rev. Lett. **121**, 145001. (10.1103/PhysRevLett.121.145001) 30339443

[47] Pribram-Jones A, Grabowski PE, Burke K. 2016 Thermal density functional theory: time-dependent linear response and approximate functionals from the fluctuation-dissipation theorem. Phys. Rev. Lett. **116**, 233001. (10.1103/PhysRevLett.116.233001) 27341227

[48] Moldabekov ZA, Dornheim T, Gregori G, Graziani F, Bonitz M, Cangi A. 2022 Towards a quantum fluid theory of correlated many-fermion systems from first principles. SciPost Phys. **12**, 062. (10.21468/SciPostPhys.12.2.062)

[49] Bethkenhagen M, Sharma A, Suryanarayana P, Pask JE, Sadigh B, Hamel S. 2023 Properties of carbon up to 10 million kelvin from Kohn-Sham density functional theory molecular dynamics. Phys. Rev. E **107**, 015306. (10.1103/PhysRevE.107.015306) 36797894

[50] Moldabekov Z, Vorberger J, Dornheim T. 2022 Density functional theory perspective on the nonlinear response of correlated electrons across temperature regimes. J. Chem. Theory Comput. **18**, 2900-2912. (10.1021/acs.jctc.2c00012) 35484932 PMC9097288

[51] Fiedler L, Moldabekov ZA, Shao X, Jiang K, Dornheim T, Pavanello M, Cangi A. 2022 Accelerating equilibration in first-principles molecular dynamics with orbital-free density functional theory. Phys. Rev. Res. **4**, 043033. (10.1103/PhysRevResearch.4.043033)

[52] Adrian PJ *et al.* 2022 Measurements of ion-electron energy-transfer cross section in high-energy-density plasmas. Phys. Rev. E **106**, L053201. (10.1103/PhysRevE.106.L053201) 36559377

[53] Falk K. 2018 Experimental methods for warm dense matter research. High Power Laser Sci. Eng. **6**, e59. (10.1017/hpl.2018.53)

[54] Glenzer SH, Redmer R. 2009 X-ray Thomson scattering in high energy density plasmas. Rev. Mod. Phys **81**, 1625. (10.1103/RevModPhys.81.1625)

[55] Kraus D *et al.* 2019 Characterizing the ionization potential depression in dense carbon plasmas with high-precision spectrally resolved x-ray scattering. Plasma Phys. Control Fusion **61**, 014015. (10.1088/1361-6587/aadd6c)

[56] MacDonald MJ *et al.* 2021 Demonstration of a laser-driven, narrow spectral bandwidth x-ray source for collective x-ray scattering experiments. Phys. Plasmas **28**, 032708. (10.1063/5.0030958)

[57] Bostedt C *et al.* 2016 Linac coherent light source: the first five years. Rev. Mod. Phys. **88**, 015007. (10.1103/RevModPhys.88.015007)

[58] Pile D. 2011 First light from SACLA. Nat. Photonics **5**, 456-457. (10.1038/nphoton.2011.178)

[59] Tschentscher T, Bressler C, Grünert J, Madsen A, Mancuso AP, Meyer M, Scherz A, Sinn H, Zastrau U. 2017 Photon beam transport and scientific instruments at the European XFEL. Appl. Sci. **7**, 592. (10.3390/app7060592)

[60] Gregori G, Glenzer SH, Rozmus W, Lee RW, Landen OL. 2003 Theoretical model of x-ray scattering as a dense matter probe. Phys. Rev. E **67**, 026412. (10.1103/PhysRevE.67.026412) 12636827

[61] Chihara J. 1987 Difference in X-ray scattering between metallic and non-metallic liquids due to conduction electrons. J. Phys. F: Met. Phys. **17**, 295-304. (10.1088/0305-4608/17/2/002)

[62] Dornheim T, Böhme M, Kraus D, Döppner T, Preston TR, Moldabekov ZA, Vorberger J. 2022 Accurate temperature diagnostics for matter under extreme conditions. Nat. Commun. **13**, 7911. (10.1038/s41467-022-35578-7) 36564411 PMC9789064

[63] Dornheim T et al. 2022 Electronic density response of warm dense matter. Phys. Plasmas **30**, 032705. (10.48550/ARXIV.2212.08326) 32909774

[64] Dornheim T, Moldabekov Z, Tolias P, Böhme M, Vorberger J. 2022 Physical insights from imaginary-time density–density correlation functions. (10.48550/ARXIV.2209.02254)

[65] Dornheim T, Moldabekov ZA, Vorberger J. 2021 Nonlinear density response from imaginary-time correlation functions: ab initio path integral Monte Carlo simulations of the warm dense electron gas. J. Chem. Phys. **155**, 054110. (10.1063/5.0058988) 34364322

[66] Dornheim T, Moldabekov Z, Vorberger J, Kählert H, Bonitz M. 2022 Electronic pair alignment and roton feature in the warm dense electron gas. Commun. Phys. **5**, 304. (10.1038/s42005-022-01078-9)

[67] Loos PF, Gill PMW. 2016 The uniform electron gas. Comput. Mol. Sci. **6**, 410-429. (10.1002/wcms.1257)

[68] Karasiev VV, Trickey SB, Dufty JW. 2019 Status of free-energy representations for the homogeneous electron gas. Phys. Rev. B **99**, 195134. (10.1103/PhysRevB.99.195134)

[69] Dornheim T. 2019 Fermion sign problem in path integral Monte Carlo simulations: quantum dots, ultracold atoms, and warm dense matter. Phys. Rev. E **100**, 023307. (10.1103/PhysRevE.100.023307) 31574603

[70] Dornheim T, Böhme M, Militzer B, Vorberger J. 2021 Ab initio path integral Monte Carlo approach to the momentum distribution of the uniform electron gas at finite temperature without fixed nodes. Phys. Rev. B **103**, 205142. (10.1103/PhysRevB.103.205142)

[71] Fosdick LD, Jordan HF. 1966 Path-integral calculation of the two-particle Slater sum for ${\mathrm{He}}^{4}$. Phys. Rev. **143**, 58-66. (10.1103/PhysRev.143.58)

[72] Jordan HF, Fosdick LD. 1968 Three-particle effects in the pair distribution function for ${\mathrm{He}}^{4}$ Gas. Phys. Rev. **171**, 128-149. (10.1103/PhysRev.171.128)

[73] Herman MF, Bruskin EJ, Berne BJ. 1982 On path integral Monte Carlo simulations. J. Chem. Phys. **76**, 5150-5155. (10.1063/1.442815)

[74] Pollock EL, Ceperley DM. 1984 Simulation of quantum many-body systems by path-integral methods. Phys. Rev. B **30**, 2555-2568. (10.1103/PhysRevB.30.2555)

[75] Takahashi M, Imada M. 1984 Monte Carlo calculation of quantum systems. J. Phys. Soc. Jpn. **53**, 963-974. (10.1143/JPSJ.53.963)

[76] Ceperley DM. 1995 Path integrals in the theory of condensed helium. Rev. Mod. Phys **67**, 279. (10.1103/RevModPhys.67.279)

[77] Chandler D, Wolynes PG. 1981 Exploiting the isomorphism between quantum theory and classical statistical mechanics of polyatomic fluids. J. Chem. Phys. **74**, 4078-4095. (10.1063/1.441588)

[78] Dornheim T, Groth S, Filinov AV, Bonitz M. 2019 Path integral Monte Carlo simulation of degenerate electrons: permutation-cycle properties. J. Chem. Phys. **151**, 014108. (10.1063/1.5093171) 31272157

[79] Loh EY, Gubernatis JE, Scalettar RT, White SR, Scalapino DJ, Sugar RL. 1990 Sign problem in the numerical simulation of many-electron systems. Phys. Rev. B **41**, 9301-9307. (10.1103/PhysRevB.41.9301) 9993272

[80] Troyer M, Wiese UJ. 2005 Computational complexity and fundamental limitations to fermionic quantum Monte Carlo Simulations. Phys. Rev. Lett. **94**, 170201. (10.1103/PhysRevLett.94.170201) 15904269

[81] Dornheim T. 2021 Fermion sign problem in path integral Monte Carlo simulations: grand-canonical ensemble. J. Phys. A: Math. Theor. **54**, 335001. (10.1088/1751-8121/ac1481)

[82] Kleinert H. 2009 Path integrals in quantum mechanics, statistics, polymer physics, and financial markets. EBL-Schweitzer. Singapore: World Scientific.

[83] Takahashi M, Imada M. 1984 Monte Carlo calculation of quantum systems. II. Higher order correction. J. Phys. Soc. Jpn. **53**, 3765-3769. (10.1143/JPSJ.53.3765)

[84] Brualla L, Sakkos K, Boronat J, Casulleras J. 2004 Higher order and infinite Trotter-number extrapolations in path integral Monte Carlo. J. Chem. Phys. **121**, 636-643. (10.1063/1.1760512) 15260589

[85] Sakkos K, Casulleras J, Boronat J. 2009 High order Chin actions in path integral Monte Carlo. J. Chem. Phys. **130**, 204109. (10.1063/1.3143522) 19485439

[86] Zillich RE, Mayrhofer JM, Chin SA. 2010 Extrapolated high-order propagators for path integral Monte Carlo simulations. J. Chem. Phys. **132**, 044103. (10.1063/1.3297888) 20113015

[87] Metropolis N, Rosenbluth AW, Rosenbluth MN, Teller AH, Teller E. 1953 Equation of state calculations by fast computing machines. J. Chem. Phys. **21**, 1087-1092. (10.1063/1.1699114) 41342507

[88] Chib S, Greenberg E. 1995 Understanding the Metropolis-Hastings algorithm. Am. Stat. **49**, 327-335. (10.1080/00031305.1995.10476177)

[89] Dornheim T, Thomsen H, Ludwig P, Filinov A, Bonitz M. 2016 Analyzing quantum correlations made simple. Contrib. Plasma Phys. **56**, 371-379. (10.1002/ctpp.201500120)

[90] Boninsegni M, Prokofev NV, Svistunov BV. 2006a Worm algorithm and diagrammatic Monte Carlo: a new approach to continuous-space path integral Monte Carlo simulations. Phys. Rev. E **74**, 036701. (10.1103/PhysRevE.74.036701) 17025780

[91] Boninsegni M, Prokofev NV, Svistunov BV. 2006b Worm algorithm for continuous-space path integral Monte Carlo Simulations. Phys. Rev. Lett **96**, 070601. (10.1103/PhysRevLett.96.070601) 16606070

[92] Chin SA. 2015 High-order path-integral Monte Carlo methods for solving quantum dot problems. Phys. Rev. E **91**, 031301. (10.1103/PhysRevE.91.031301) 25871047

[93] Dornheim T, Groth S, Filinov A, Bonitz M. 2015 Permutation blocking path integral Monte Carlo: a highly efficient approach to the simulation of strongly degenerate non-ideal fermions. New J. Phys. **17**, 073017. (10.1088/1367-2630/17/7/073017)

[94] Dornheim T, Groth S, Bonitz M. 2019 Permutation blocking path integral Monte Carlo simulations of degenerate electrons at finite temperature. Contrib. Plasma Phys. **59**, e201800157. (10.1002/ctpp.201800157)

[95] Filinov A, Levashov PR, Bonitz M. 2021 Thermodynamics of the uniform electron gas: fermionic path integral Monte Carlo simulations in the restricted grand canonical ensemble. Contrib. Plasma Phys. **61**, e202100112. (10.1002/ctpp.202100112)

[96] Jarrell M, Gubernatis J. 1996 Bayesian inference and the analytic continuation of imaginary-time quantum Monte Carlo data. Phys. Rep. **269**, 133-195. (10.1016/0370-1573(95)00074-7)

[97] Dornheim T, Groth S, Vorberger J, Bonitz M. 2018 Ab initio path integral Monte Carlo results for the dynamic structure factor of correlated electrons: from the electron liquid to warm dense matter. Phys. Rev. Lett. **121**, 255001. (10.1103/PhysRevLett.121.255001) 30608805

[98] Groth S, Dornheim T, Vorberger J. 2019 Ab initio path integral Monte Carlo approach to the static and dynamic density response of the uniform electron gas. Phys. Rev. B **99**, 235122. (10.1103/PhysRevB.99.235122) 28950530

[99] Fraser LM, Foulkes WMC, Rajagopal G, Needs RJ, Kenny SD, Williamson AJ. 1996 Finite-size effects and Coulomb interactions in quantum Monte Carlo calculations for homogeneous systems with periodic boundary conditions. Phys. Rev. B **53**, 1814-1832. (10.1103/PhysRevB.53.1814) 9983641

[100] Kugler AA. 1975 Theory of the local field correction in an electron gas. J. Stat. Phys **12**, 35-87. (10.1007/BF01024183)

[101] Marques M, Maitra N, Nogueira F, Gross E, Rubio A 2012 Fundamentals of time-dependent density functional theory. Lecture Notes in Physics. Berlin, Germany: Springer.

[102] Senatore G, Moroni S, Ceperley DM. 1996 Local field factor and effective potentials in liquid metals. J. Non-Cryst. Sol. **205–207**, 851-854. (10.1016/S0022-3093(96)00316-X)

[103] Moldabekov Z, Groth S, Dornheim T, Kählert H, Bonitz M, Ramazanov TS. 2018 Structural characteristics of strongly coupled ions in a dense quantum plasma. Phys. Rev. E **98**, 023207. (10.1103/PhysRevE.98.023207) 30253556

[104] Moldabekov Z, Kählert H, Dornheim T, Groth S, Bonitz M, Ramazanov TS. 2019 Dynamical structure factor of strongly coupled ions in a dense quantum plasma. Phys. Rev. E **99**, 053203. (10.1103/PhysRevE.99.053203) 31212426

[105] Moldabekov ZA, Bonitz M, Ramazanov TS. 2018 Theoretical foundations of quantum hydrodynamics for plasmas. Phys. Plasmas **25**, 031903. (10.1063/1.5003910)

[106] Moldabekov Z, Bonitz M, Ramazanov T. 2017 Gradient correction and Bohm potential for two- and one-dimensional electron gases at a finite temperature. Contrib. Plasma Phys. **57**, 499-505. (10.1002/ctpp.201700113)

[107] Patrick CE, Thygesen KS. 2015 Adiabatic-connection fluctuation-dissipation DFT for the structural properties of solids—the renormalized ALDA and electron gas kernels. J. Chem. Phys. **143**, 102802. (10.1063/1.4919236) 26373995

[108] Singwi KS, Tosi MP, Land RH, Sjölander A. 1968 Electron correlations at metallic densities. Phys. Rev. **176**, 589. (10.1103/PhysRev.176.589)

[109] Vashishta P, Singwi KS. 1972 Electron correlations at metallic densities V. Phys. Rev. B **6**, 875. (10.1103/PhysRevB.6.875)

[110] Ichimaru S, Iyetomi H, Tanaka S. 1987 Statistical physics of dense plasmas: thermodynamics, transport coefficients and dynamic correlations. Phys. Rep. **149**, 91-205. (10.1016/0370-1573(87)90125-6)

[111] Schweng HK, Böhm HM. 1993 Finite-temperature electron correlations in the framework of a dynamic local-field correction. Phys. Rev. B **48**, 2037. (10.1103/PhysRevB.48.2037) 10008593

[112] Farid B, Heine V, Engel GE, Robertson IJ. 1993 Extremal properties of the Harris-Foulkes functional and an improved screening calculation for the electron gas. Phys. Rev. B **48**, 11602. (10.1103/PhysRevB.48.11602) 10007497

[113] Stolzmann W, Rösler M. 2001 Static local-field corrected dielectric and thermodynamic functions. Contrib. Plasma Phys. **41**, 203-206. (10.1002/1521-3986(200103)41:2/3<203::AID-CTPP203>3.0.CO;2-S)

[114] Dabrowski B. 1986 Dynamical local-field factor in the response function of an electron gas. Phys. Rev. B **34**, 4989-4995. (10.1103/PhysRevB.34.4989) 9940320

[115] Holas A, Rahman S. 1987 Dynamic local-field factor of an electron liquid in the quantum versions of the Singwi-Tosi-Land-Sjölander and Vashishta-Singwi theories. Phys. Rev. B **35**, 2720-2731. (10.1103/PhysRevB.35.2720) 9941748

[116] Arora P, Kumar K, Moudgil RK. 2017 Spin-resolved correlations in the warm-dense homogeneous electron gas. Eur. Phys. J. B **90**, 76. (10.1140/epjb/e2017-70532-y)

[117] Tanaka S. 2017 Improved equation of state for finite-temperature spin-polarized electron liquids on the basis of Singwi–Tosi–Land–Sjölander approximation. Contrib. Plasma Phys. **57**, 126-136. (10.1002/ctpp.201600096)

[118] Tanaka S. 2016 Correlational and thermodynamic properties of finite-temperature electron liquids in the hypernetted-chain approximation. J. Chem. Phys. **145**, 214104. (10.1063/1.4969071) 28799346

[119] Tolias P, Lucco Castello F, Dornheim T. 2021 Integral equation theory based dielectric scheme for strongly coupled electron liquids. J. Chem. Phys. **155**, 134115. (10.1063/5.0065988) 34625000

[120] Castello FL, Tolias P, Dornheim T. 2022 Classical bridge functions in classical and quantum plasma liquids. Europhys. Lett. **138**, 44003. (10.1209/0295-5075/ac7166)

[121] Hamann P, Dornheim T, Vorberger J, Moldabekov ZA, Bonitz M. 2020 Dynamic properties of the warm dense electron gas based on ab initio path integral Monte Carlo simulations. Phys. Rev. B **102**, 125150. (10.1103/PhysRevB.102.125150)

[122] Dornheim T, Vorberger J. 2020 Finite-size effects in the reconstruction of dynamic properties from ab initio path integral Monte Carlo simulations. Phys. Rev. E **102**, 063301. (10.1103/PhysRevE.102.063301) 33466040

[123] Böhme M, Moldabekov ZA, Vorberger J, Dornheim T. 2022 Static electronic density response of warm dense hydrogen: ab initio path integral Monte Carlo simulations. Phys. Rev. Lett. **129**, 066402. (10.1103/PhysRevLett.129.066402) 36018668

[124] Böhme M, Moldabekov ZA, Vorberger J, Dornheim T. 2022 Ab initio path integral Monte Carlo simulations of hydrogen snapshots at warm dense matter conditions. Phys. Rev. E **107**, 015206. (10.48550/ARXIV.2207.14716) 36797933

[125] Moldabekov ZA, Böhme M, Vorberger J, Blaschke D, Dornheim T. 2023 Ab initio static exchange–correlation kernel across Jacob’s ladder without functional derivatives. J. Chem. Theory Comput. **19**, 1286-1299. (10.1021/acs.jctc.2c01180) 36724889 PMC9979610

[126] Bowen C, Sugiyama G, Alder BJ. 1994 Static dielectric response of the electron gas. Phys. Rev. B **50**, 14838. (10.1103/PhysRevB.50.14838) 9975827

[127] Dornheim T, Vorberger J, Groth S, Hoffmann N, Moldabekov Z, Bonitz M. 2019 The static local field correction of the warm dense electron gas: an ab initio path integral Monte Carlo study and machine learning representation. J. Chem. Phys. **151**, 194104. (10.1063/1.5123013) 31757143

[128] Dornheim T, Sjostrom T, Tanaka S, Vorberger J. 2020 Strongly coupled electron liquid: ab initio path integral Monte Carlo simulations and dielectric theories. Phys. Rev. B **101**, 045129. (10.1103/PhysRevB.101.045129)

[129] Dornheim T, Moldabekov ZA, Vorberger J, Groth S. 2020 Ab initio path integral Monte Carlo simulation of the uniform electron gas in the high energy density regime. Plasma Phys. Controlled Fusion **62**, 075003. (10.1088/1361-6587/ab8bb4)

[130] Mihara N, Puff RD. 1968 Liquid structure factor of ground-state ${\mathrm{He}}^{4}$. Phys. Rev. **174**, 221-227. (10.1103/PhysRev.174.221)

[131] Tkachenko IM, Arkhipov YV, Askaruly A. 2012 The method of moments and its applications in plasma physics. Saarbrücken, Germany: Akademikerverlag.

[132] Dornheim T, Tolias P, Moldabekov ZA, Cangi A, Vorberger J. 2022 Effective electronic forces and potentials from ab initio path integral Monte Carlo simulations. J. Chem. Phys. **156**, 244113. (10.1063/5.0097768) 35778089

[133] Dornheim T, Vorberger J, Moldabekov ZA, Tolias P. 2022 Spin-resolved density response of the warm dense electron gas. Phys. Rev. Res. **4**, 033018. (10.1103/PhysRevResearch.4.033018)

[134] Dornheim T, Moldabekov ZA, Tolias P. 2021 Analytical representation of the local field correction of the uniform electron gas within the effective static approximation. Phys. Rev. B **103**, 165102. (10.1103/PhysRevB.103.165102)

[135] Kugler AA. 1970 Bounds for some equilibrium properties of an electron gas. Phys. Rev. A **1**, 1688. (10.1103/PhysRevA.1.1688)

[136] Hamann P, Vorberger J, Dornheim T, Moldabekov ZA, Bonitz M. 2020 Ab initio results for the plasmon dispersion and damping of the warm dense electron gas. Contrib. Plasma Phys. **60**, e202000147. (10.1002/ctpp.202000147)

[137] Baerwinkel K. 1971 Dynamic structure factor of ideal quantum systems. Physik der kondensierten Materie **12**, 287-291. (10.1007/BF02422556)

[138] Takada Y. 2016 Emergence of an excitonic collective mode in the dilute electron gas. Phys. Rev. B **94**, 245106. (10.1103/PhysRevB.94.245106)

